# The Transcription Factor ThPOK Regulates ILC3 Lineage Homeostasis and Function During Intestinal Infection

**DOI:** 10.3389/fimmu.2022.939033

**Published:** 2022-07-01

**Authors:** Xianzhi Gao, Xin Shen, Kuai Liu, Chenyu Lu, Ying Fan, Qianying Xu, Xiaoyu Meng, Shenghui Hong, Zhengwei Huang, Xia Liu, Linrong Lu, Lie Wang

**Affiliations:** ^1^ Institute of Immunology, and Bone Marrow Transplantation Center of the First Affiliated Hospital, Zhejiang University School of Medicine, Hangzhou, China; ^2^ Zhejiang University-University of Edinburgh Institute, Zhejiang University School of Medicine, Hangzhou, China; ^3^ Department of Biomedical Sciences, City University of Hong Kong, Hong Kong, China; ^4^ Laboratory Animal Center, Zhejiang University, Hangzhou, China; ^5^ Zhejiang University School of Medicine, Hangzhou, China; ^6^ Liangzhu Laboratory, Zhejiang University Medical Center, Hangzhou, China; ^7^ Zhejiang University (ZJU)-Hangzhou Global Scientific and Technological Innovation Center, Hangzhou, China; ^8^ Cancer Center, Zhejiang University, Hangzhou, China

**Keywords:** ILC3s, homeostasis, transcription factor, ThPOK, mucosa

## Abstract

Innate lymphoid cells (ILCs) have been identified as a heterogeneous population of lymphocytes that mirrors the cytokine and transcriptional profile of adaptive T cells. The dynamic balance between key transcription factors determines the heterogeneity, plasticity, and functions of ILC subsets. The transcription factor ThPOK is highly conserved in biological evolution and exerts pivotal functions in the differentiation of T cells. However, the function of ThPOK in ILC3s has not been identified. Here, we found that ThPOK regulated the homeostasis of ILC3s, as mice lacking ThPOK showed decreased NKp46^+^ ILC3s and increased CCR6^-^ NKp46^-^ ILC3s. ThPOK-deficient mice were more sensitive to *S. typhimurium* infection due to the impaired IFN-γ secretion of NKp46^+^ ILC3s. Furthermore, ThPOK participates in ILC3-mediated control of *C. rodentium* infection by negatively regulating IL-17A secretion. ThPOK preserves the identity of NKp46^+^ ILC3s by repressing RORγt, which indirectly releases T-bet expression. On the molecular level, ThPOK directly binds to *Rorc* and *Il23r* to restrain their expression which further modulates IL-17A secretion. Collectively, our analysis revealed a critical role of ThPOK in the homeostasis and functions of ILC3 subsets.

**Graphical Abstract:**
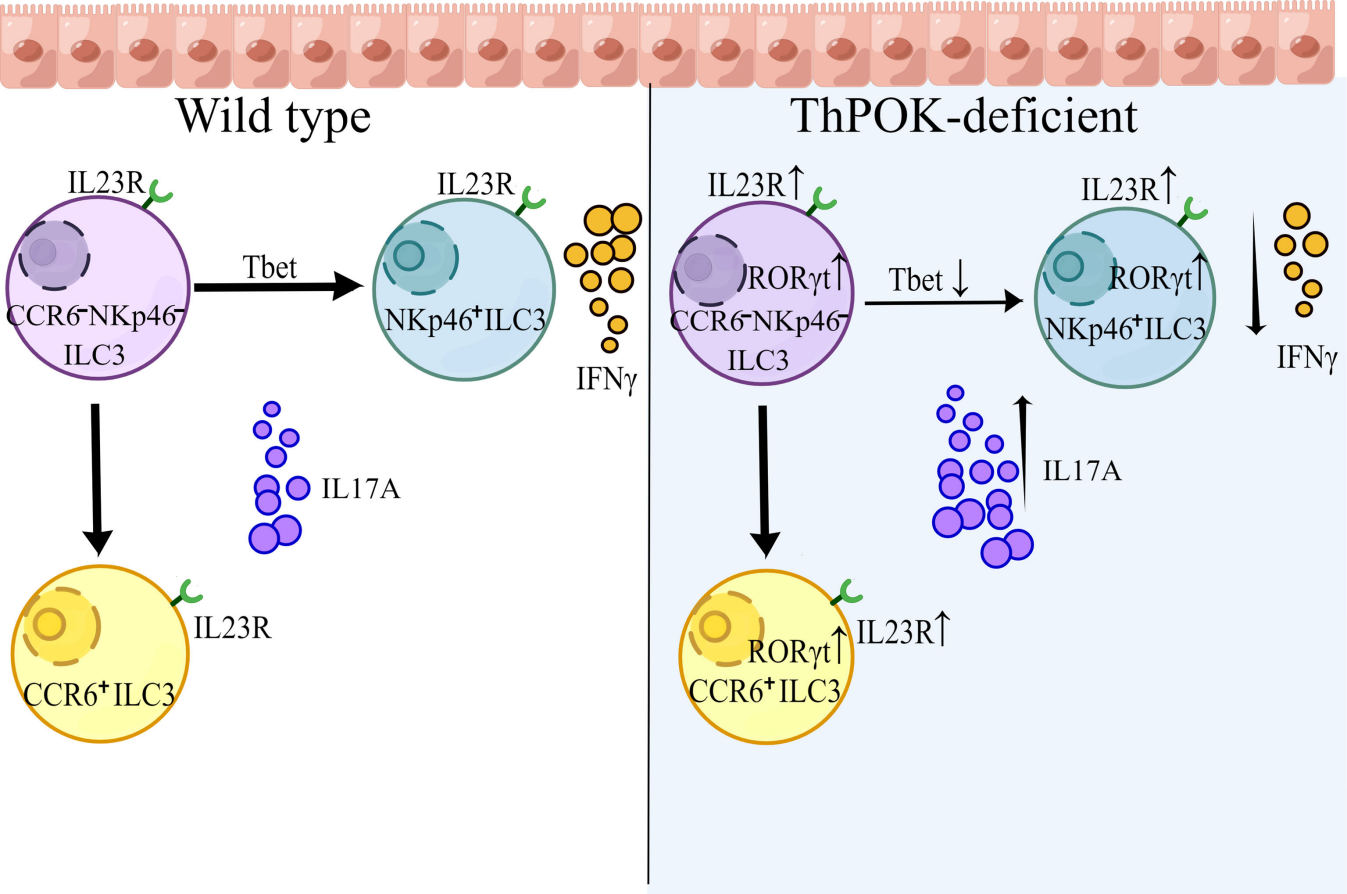


## Introduction

Innate lymphoid cells are enriched at mucosal surfaces, representing the first line of host defense against bacterial infections (1). They develop from common lymphoid precursors (CLPs) that also differentiate into to adaptive cell precursors (B or T cells) (2), whereas ILCs lack the expression of genetically rearranged antigen receptors, and they do not respond to antigen presentation (3). Interestingly, subsets of ILCs mirror the characteristics of adaptive T cells in both phenotypes and functions (4, 5). According to defined functional cytokines and key transcription factors, ILCs can be phenotypically classified into three distinct subgroups. Group 1 ILCs (ILC1 cells and NK cells) constitutively express T-bet and secrete type-1 cytokines in response to intracellular bacterial infections such as IFN-γ and TNF-α (6). ILC2s are characterized by their master regulator GATA-3 and type 2 cytokines, including interleukin (IL)-4, IL-5, IL-9, and IL-13 (7). RORγt-dependent ILC3s are innate sources of IL-17A, IL-17F and IL-22 that are required for mediating immunity to extracellular bacterial infections (8–11).

As a heterogeneous population, ILC3s can be subsequently classified as CCR6^+^ ILC3s, NKp46^+^ ILC3s, and CCR6^-^ NKp46^-^ ILC3s (DN ILC3s). CCR6^+^ ILC3s comprise CD4^+^ and CD4^-^ cells, whereas some CCR6^-^ ILC3s upregulate T-bet and eventually develop into NKp46^+^ ILC3s in response to microbiomes and Notch-dependent signals (12–14). Functionally, ILC3s maintain gut epithelial integrity and combat infection by secretion of type 3 cytokines production (9, 11, 15–17). During the natural murine intestinal pathogen *C. rodentium* infection, early production of IL-22 and IL-17 from ILC3s are required for resistance (18). Moreover, IL‐17A production by ILC3s contributes to protection against fungal infection, and IL‐17A deficiency significantly weakens the ability to resist systemic dissemination of *C. albicans* in mice (17, 19). In *Salmonella Typhimurium* infection models, ILC3s accumulate in the inflamed colon, and infections increase the proportions of NKp46^+^ ILC3s and IFN-γ secretion (20, 21).

Transcription factors are indispensable for the diversity and plasticity of ILC3s. Recently, multiple transcription factors have been identified as being involved in ILC3 regulation. RORγt is strictly required for driving the differentiation of ILC3s from their precursors (22), and controls the production of Th17 cytokines IL-17 and IL-22 (23). As a ligand-activated transcription factor, aryl hydrocarbon receptor (AHR) is preferentially highly expressed in ILC3s, responsible for their maintenance and function (24, 25). The transcription factor GATA-3 has been identified to regulate the development of NKp46^+^ ILC3s and to determine the cell fate between NKp46^+^ and CCR6^+^ ILC3s by regulating the dynamic balance between T-bet and RORγt (26). Deletion of *Gata3* blocks the further transition from CCR6^-^ NKp46^-^ ILC3s to NKp46^+^ ILC3s. In addition, GATA-3 can drive the production of IL-22 in ILC3s by directly bind to the promoter of the *Il22* gene (24, 26, 27). Interestingly, a subset of CCR6^-^ ILC3s co-express RORγt and the master regulator T-bet. T-bet regulates the production of IFN-γ and a high level of T-bet serves as a repressor of RORγt. Furthermore, dynamic expression of RORγt and T-bet in NKp46^-^ ILC3s acts as a molecular switch, and is associated with ILC3 plasticity (28). One important factor is Runx3, as it can bind to the promoter of *Rorc* and induce the expression of RORγt and its downstream target AHR. Deletion of Runx3 in NKp46-expressing cells resulted in a significant decrease of NKp46^+^ ILC3s, along with attenuated control of *C. rodentium* infection (29, 30). Additionally, transcriptional analysis has shown that c-Maf restraints T-bet expression to regulate the ILC3-ILC1 balance (31, 32). However, the combined role of multiple transcription factors in ILC plasticity and the precise molecular mechanisms underlying functional integration remain unclear.

T-helper-inducing POZ-Kruppel Factor (Th-POK, also known as ZBTB7B or cKrox), is a Zn finger transcription factor belonging to the BTB-POZ family, which is characterized by an N-terminal BTB domain (33). The central role of ThPOK in lineage commitment of CD4^+^ T cells and the underling mechanism has benn well established (34). In this report, we observed that mice lacking ThPOK exhibited a selective loss of CCR6^-^ NKp46^+^ ILC3s with a concomitant increase in CCR6^-^ NKp46^-^ ILC3s. Deficiency in IFN-γ-producing NKp46^+^ ILCs resulted in greater susceptibility of ThPOK-deficient mice to infection with the intracellular bacterial pathogen *S. typhimurium*. We also found that IL-17A-producing cells were strongly increased during *C. rodentium* infection, although IL-22 production remained stable. To further elucidate the underlying mechanism, we carried out single-cell RNA sequencing. Analysis revealed that the expression of RORγt was significantly increased, disturbing the balance between RORγt and T-bet. Moreover, ThPOK directly binds to *Rorc* and *Il23r*, which modulate the IL-23/IL-17 axis to trigger IL-17A secretion. Taken together, our data reveal an unknown function of ThPOK in maintaining the functionality and lineage stability of ILC3s.

## Materials and Methods

### Mice

ThPOK conventional knockout mice were purchased from the Jackson Laboratory (Bar Harbor, ME). All the mice used in this study are on a C57BL/6 background and were maintained in a specific pathogen-free facility in the Biological Animal Center at the Zhejiang University and analyzed at 6-8 weeks of age unless otherwise described. NOD Prkdc^em26Cd^52Il2rg^em26Cd22^/Nju (NCG, T001475) mice were purchased from Nanjing Biomedical Research Institute of Nanjing University.

### Isolation of Intestinal LPLs

Intestinal lymphocytes were isolated from the small intestine of adult mice as previously described (30). Briefly, small intestines were cut open longitudinally and washed with DMEM after removing fat tissues and Peyer’s patches. The intestines were subsequently cut into pieces of equal length and width, shaken and digested in pre-warmed buffer containing DMEM, 3% FBS, 0.2% Hanks, 5 mM EDTA, and 0.145 mg/ml DTT for 10 min in the constant temperature water bath at 37°C. The detached cells from the intestinal tissues were collected as IELs. To obtain lamina propria lymphocytes, the remaining pieces of small intestine was incubated and digested in a solution of 3% DMEM, 0.2% FBS, 0.025% Hanks, 50 mg/ml DNase, and 75 mg/ml collagenase II twice (5 min every time). Then, those dissociated cells were sifted through a cell strainer (100µm) and collected for further percoll (GE Healthcare) density gradient centrifugation.

### Flow Cytometry

The following commercial antibodies were used in our experiments: CD3e (145-2C11, eBioscience), TCRγδ (GL3, eBioscience), Gr-1 (RB6-8C5, Biolegend), TER-119 (TER-119, Biolegend), CD19 (1D3, eBioscience), CD5 (53-7.3, Biolegend), B220 (RA3-6B2, Tianjin Sungene Biotech), NK1.1 (PK136, Biolegend), CD11c (N418, Biolegend), CD11b (M1/70, Biolegend), CD27 (LG.7F9, eBioscience), IL-17A (TC11-18H10, BD), CD45.1 (A20, Biolegend), IFN-γ (XMG1.2, BD), Ki67 (B56, BD), GATA-3 (TWAJ, eBioscience), CD45.2 (104, BD), RORγt (Q31-378, BD), CD127 (A7R34, eBioscience), NK1.1 (PK136, BD), CD335 (NKp46) (29A1.4, Biolegend), IL-22 (IL22JOP, eBioscience), KLRG1 (2F1, BD), C-KIT (CD117) (2B8, eBioscience), EOMES (Dan11mag, invitrogen), CCR6 (29-2L17, Biolegend), Isotype (P3.6.2.8.1, eBioscience), B220 (RA3-6B2, Biolegend), IgM (II/41, BD), CD8α (53-6.7, Biolegend), PLZF (9E12, Biolegend), CD4 (RM4-4, Biolegend), Streptavidin (BD), α4β7 (DATK32), Sca-1 (D7, Biolegend), Flt3 (A2F10, eBioscience), CD25 (PC61.5, eBioscience), TCR-β (H57-597, Biolegend), TCF-7 (C6309, CST). FITC annexin V (1:20 dilution) was purchased from BioLegend. We use IC fixation buffer (eBiosciences) for intracellular staining. For flow cytometry, cells were stained with primary antibodies for 0.5 h at room temperature, followed by fix, permeabilization, and nuclear staining of transcription factors. ILCs were defined as Lineage^-^ cells (lineage markers included TCRγδ, CD3ϵ, CD19, CD5, Gr-1 and Ter119). ILC1s were gated as Lin^-^ NKp1.1^+^ NKp46^+^ Eomes^-^ RORγt^-^. NK cells were gated as Lin^-^ NKp1.1^+^ NKp46^+^ Eomes^+^ RORγt^-^. ILC2s were gated as Lin^-^ CD127^-^ KLRG1^+^ GATA3^+^. CCR6^+^ ILC3s were gated as Lin^-^ RORγt^+^ CCR6^+^ NKp46^-^. NKp46^+^ ILC3s were gated as Lin^-^ RORγt^+^ CCR6^-^ NKp46^+^. CCR6^-^ NKp46^-^ ILC3s were gated as Lin^-^ RORγt^+^ CCR6^-^ NKp46^-^. Flow cytometry was performed with BD Fortessa (BD Biosciences). Data were analyzed with FlowJo™10 software. Cells were sorted by a FACS Aria II flow cytometer.

### S. typhimurium Infection

Before *S. typhimurium* intestinal infection, we fasted mice for 4 h and administer 20 mg streptomycin *via* gavage. After 20 h, 10^9^ *S. typhimurium* (SL1344, SB300) was taken to infect mice *via* oral gavage. 4 days after the infection, the infected mice were sacrificed for the following experiments. The small intestines were collected to isolate intestinal lymphocyte cells. In addition, the colon was fixed in 4% methanol for H&E staining. Weight loss was measured daily for further analysis and those data were normalized to the mass recorded on the day of infection.

### C. rodentium Infection

*C. rodentium* was obtained from Dr. Ju Qiu (Shanghai Institute of Nutrition and Health, Shanghai Institutes for Biological Sciences, Chinese Academy of Sciences). Co-housed experimental mice were orally infected with 2 × 10^9^ colony forming units (CFU) of *C. rodentium* in PBS. We keep recording the weight loss in the following days. On day 8 post-infection, mice were sacrificed to examine colon pathology and bacterial loads. Feces were collected to be weighed and plated to determine the CFU. The small intestines were collected and used to isolate LPLs and the colon was fixed in 4% methanol for H&E staining.

### *Candida albicans* Infection

*Candida albicans* was obtained from Dr. Guanghua Huang (Fudan University). To induce *Candida albicans* infection, mice were administered with drinking water containing 2 mg/ml Streptomycin, 0.2 mg/ml Fluconazole and 0.2 mg/ml Gentamicin for 2 days. From the third day, treated mice with drinking water containing 2 mg/ml Streptomycin, 0.2 mg/ml Gentamicin, and 2 mg/ml Ampicillin to the last. On the fourth day, mice were orally infected with *Candida albicans* (2 × 10^8^ per mouse), followed by recording of survival rate and body change in the following days. 10 days post infection, mice were sacrificed to examine colon pathology and bacterial loads.

### Adopt-Transplantation of ILC to NCG Mice

80000 intestinal NKp46^+^ ILC3s (CD3^-^/CD19^-^/KLRG1^-^/CD127^-^/RORgt^+^/NKp46^+^) were purified from the siLP of ThPOK-deficient mice and their WT littermates or PBS by a FACS Aria III sorter (BD). Sorted cells were stimulated with IL-12 and IL-18 at 40 ng/mL for an hour to support cell viability and optimal IFN-γ production before injection into NCG mice through the tail vein. The recipient NCG mice were infected with *S. typhimurium* 24 hours post adoptive transfer. Body weight were monitored for the following days, and mice were sacrificed for further analysis at day 9.

NCG mice were adoptively transferred with 80000 intestinal ILC3s (CD3^-^/CD19^-^/KLRG1^-^/CD127^-^/RORgt^+^) sorted from ThPOK-deficient mice and their WT littermates or PBS as control after treated with ABX for 1 week. ILC3s were stimulated with IL-23 and IL-1β, at the concentration of 40 ng/ml, for 30 min before injected to NCG mice through the tail vein. NCG mice were orally inoculated with *C. rodentium* 24 hours post adoptive transfer. Body weight were monitored for the following days, and all mice were sacrificed at day 9.

### Generation of Cells Constitutively Express 3xflag-ThPOK

We used EL4 (ATCC^®^ TIB-39™) cell line. EL4 and Plat-E were cultured in DMEM with 10% FBS and 1% antibiotics. All cultures were keep at 37°C in a cell incubator, with 95% humidity and 5% CO_2_-air. Retrovirus preparation was performed in suitable Plat-E cells and transfected with pMX-IRES-GFP containing 3xflag tagged ThPOK gene. After 10 h, the medium was replaced with fresh medium. After an additional 72 h, the retrovirus supernatant was collected and storage. EL4 cells were suspended in 1 ml of virus supernatant with 8 μg/ml polybrene (Sigma) at 2,500g for at least 2 h at 32°C. After 24 h, the retroviral transduction was repeated. Five days post transduction, sorting GFP positive cells for further analysis, which constitutively express flag labeled ThPOK.

### ChIP-qPCR

Sort cells by flow cytometry directly in a 1.5 ml sample tube. Seeding cells in Nuclei Extraction Buffer. According to the input size, chromatin was fragmented for 5-7.5 min using MNase at 37 °C, and diluted in 10X MNase Storage Buffer (MNase master buffer (BioLabs), 100 mM DTT, 50% PEG6000, 100 × BSA, 10 U/μl MNase (BioLabs). Proceed at room temperature (25°C) for 5 min and add a 5.5μl MNase Stop Solution (100 mM EDTA). Add 5.5 μl Nuclear Break Buffer (1% Triton, 1% DOC solution) into the tube, mix well by the gentle vortex. These are sheared chromatin ready to be ChIPed. Chromatin was pre-cleared with 5μl of 1:1 protein A: G Dynabeads (Life Technologies) with 0.25 mg of Flag (Cell Signaling Technology No. D6W5B) antibody-bead complexes overnight at 4 °C. ChIPed complexes were washed twice with 100 μl of ice-cold Low Salt Wash Buffer (10 mM Tris-HCl, pH 8.0, 0.1% SDS, 1% Triton X-100, 2 mM EDTA, 150 mM NaCl, 1× PIC) and twice with High Salt Wash Buffer (10 mM Tris-HCl, pH 8.0, 0.1% SDS, 1% Triton X-100, 2 mM EDTA, 150 mM NaCl, 1× PIC). ChIPed material was purified by equal volume phenol: chloroform: isoamyl alcohol into the PhaseLock tube. Add 1/10 of the vol. (10 μl if use 100 μl) 3M NaOAc and 1μl of LPA. Protein-DNA complexes were eluted in 30 μl of DNA elution buffer.

### Single-Cell RNA-Sequencing

All samples were sorted from siLP by FACS Aria III sorter (BD) and washed twice by pre-cooled BD Pharmingen Stain Buffer (FBS). Briefly centrifuge and re-suspend cells in 200 µL stain buffer to prepare a single cell suspension. For each sample, transfer 180 µL cell suspension to a Sample Tag tube (BD Mouse Single-Cell Multiplexing Kit, USA). Then cells for all samples were labeled with sample tags, incubated, counted, and multiplexed. Up to 4 barcoded samples were pooled and ready for capture with cell capture beads. Single-cell capture and cDNA synthesis were performed by the BD Rhapsody Single-Cell Analysis System (BD Biosciences). cDNA libraries were prepared using the BD Rhapsody Whole Transcriptome Analysis Amplification Kit according to the manufacturer’s recommendations (BD Biosciences). The final libraries were quantified with Agilent 2100-H and processed on multiple runs of Illumina NovaSeq 6000 for sequencing.

### Analysis of scRNA-Seq Data

The clean data of the FASTQ files were uploaded and processed using the BD Rhapsody Targeted analysis pipeline v1.9.1 on the Seven Bridges platform (https://www.sevenbridges.com). First of all, read pairs with low mean base quality scores (less than 20) were removed. The filtered R1 reads were analyzed to identify the cell label sequences and unique molecular identifiers (UMIs). R2 reads were aligned to the mice reference genome (GRCm38) using Bowtie2. Valid reads with the same cell label, the same UMI sequence, and the same gene were collapsed into a single raw molecule. Recursive substation error correction (RSEC) and distribution-based error correction (DBEC) algorithms were applied to correct sequencing and PCR errors of raw UMI counts. The final single-cell expression matrices containing DBEC-adjusted molecules were used for downstream analysis.

### Unsupervised Clustering of Cells and T-Distributed Stochastic Neighbor Embedding-Visualization

Quality control and downstream analysis of gene expression matrix were performed using Seurat v4.03. The expression matrix was log normalized and scaled taking into account UMI counts. All targeted genes were used for Principal components analysis (PCA). The PCA matrix was then fed into HarmonyMatrix() function in the Harmony R package (Korsunsky et al., 2019) to integrate different samples. Clusters were identified by shared nearest neighbor (SNN) based clustering and visualized using Manifold Approximation and Projection (UMAP). A method mentioned in Ruiping Wang’s paper (Ruiping Wang et al., 2021) was used to remove doublets. Specifically, if cells of a certain cluster express markers of different lineages, this cluster will be discarded. Possible cell doublets were removed from each cluster after the initial clustering and the remaining single cells were re-clustered again. With the data of SC-sequencing, all unsupervised clustering of cells was performed with R (Seurat package version 2.2). Particularly, Genes were kick out if these were expressed in less than two cells. Cells with >200 genes and <10% of mitochondrial genes were gathered for further processoin. Then, calculate the variation coefficient of genes with Seurat arithmetic. With the first 1,500 highest alterable genes, principle component analysis (PCA) was used to perform dimensionality reduction of all data. Then the results of unsupervised clustering were visualized by using the tSNE project. Cells with high expression of mitochondrial genes were abandoned.

### Cell Developmental Trajectory Analysis

The Monocle2 (Qiu et al., 2017) was used to perform pseudo-time trajectories analysis in ILC cells independently. First of all, we took use of ‘‘relative2abs’’ function in Monocle2 to convert TPM into normalized mRNA counts and then created an object with parameter ‘‘expression Family = neg-binomial. size’’ following the Monocle2 tutorial. We used the ‘‘differentialGeneTest’’ function to derive DEG from each cluster and genes with a q-value < 1e-5 were used to order the cells in pseudo-time analysis. After constructing the cell trajectories, we used the ‘‘differentialGeneTest’’ function to detect differentially expressed genes along the pseudo time. Differential genes (DEGs) between the clusters were selected for PCA-based dimension reduction and following visualization on UMAP. Naïve cells clustered on the UMAP were set as the root state for calculating distinct lineages and pseudo-time.

### Bioinformatic Analysis of RNA-Seq Data

The sequencing data was filtered with SOAPnuke (v1.5.2) by removing reads containing sequencing adapter. Then, removing reads whose low-quality base ratio (base quality less than or equal to 5) is more than 20%. Last, removing reads whose unknown base (‘N’ base) ratio is more than 5% and afterwards clean reads were obtained and stored in FASTQ format. Mapping the clean reads to the reference genome with HISAT2 (v2.0.4). Applying the Bowtie2 (v2.2.5) to align the clean reads to the reference gene set. Then, the expression level of gene was calculated *via* RSEM (v1.2.12). Particularly, the differential expression analysis was performed by using the DESeq2(v1.4.5) with Q value ≤ 0.05. GO (http://www.geneontology.org/) and KEGG (https://www.kegg.jp/) enrichment analysis of annotated different expressed gene was performed by Phyper (https://en.wikipedia.org/wiki/Hypergeometric_distribution) based on Hypergeometric test. The significant levels of terms and pathways were corrected by Q value with a rigorous threshold (Q value ≤ 0.05) by Bonferroni. The differentially expressed genes of intestinal ILC3 cells from wile-type and ThPOK-deficient mice were analyzed and draw volcano plots online at https://www.omicstudio.cn/tool.

### Luciferase Reporter Assay

To form a luciferase reporter plasmid, the introns 2 and 3 of *Rorc*, intron 2 of *Il23r* and their mutants were generated by PCR amplification and subsequently subcloned into the Vector (pGL3-Enhancer). Human embryonic kidney (HEK293) cells were co-transfected with 100 ng of the luciferase reporter plasmid, 10 ng of a thymidine kinase promoter-Renilla luciferase reporter plasmid, plus the pCDNA3-ThPOK or control vector. Following the manufacturer’s instructions, the luciferase activities were detected by the Dual-Luciferase Reporter Assay System (Promega, Cat. No. E10910) 48 h after transfection. Luciferase activity was measured by 1450 Micro Beta (Wallac).

The primers were as followed:

IL23RA: CGCGGATCCTCTAGATACATAGATAGAAGATAGATGGT
CCCAAGCTTAGAAGGGATGGGATGTGAGCTTTGTAT
RORγt-A: CCGGAATTCCATAGAGAGAAGCTATTC
CCGCTCGAGTCAGCCTTGGAGGTTCCT
RORγt-B: CCCAAGCTTGTGTCATAGACTGGCTTTG
CCGCTCGAGACTGACATACCTGATATC

We synthesized the whole gene fragment of RORγt-A (mut), RORγt-B (mut) and IL23R (mut) by mutating the ThPOK binding site GGGAGGG into TTGCAAG.

### Plasmid Constructs

The vector encoding mouse ThPOK was constructed by PCR amplification and subcloned into the pcDNA3 eukaryotic expression vector. Recombinant vectors encoding mouse 3xflag-ThPOK, *Rorc*, and *Il23r* were subcloned into the pMX-IRES-GFP eukaryotic expression vector.

### Cytokine Stimulation

T cells were sorted and seeded in a 96-well plate. Stimulating the cells with PMA (25 ng/ml) to analyze the intracellular INF-γ. Stimulating with PMA (25 ng/ml) and ionomycin (500 ng/ml) to analyze the intracellular TNF-α. Cells were stimulated with PMA (0.1 μg/ml, Sigma-Aldrich), ionomycin (0.5 μg/ml, Sigma-Aldrich) in the presence of Brefeldin A (10 μg/ml, Sigma-Aldrich) For IL-17A intracellular cytokine staining. For IL-22 production, adding IL-23 (40 ng/ml, R&D Systems, Minneapolis, MN), PMA, ionomycin, and Brefeldin A into cultures for 4 h. Cells were cultured at 37°C in DMEM medium supplemented with 20% FBS for 4 h. Following the manufacturer’s instructions, cells were fixed and permeabilized with kits for intracellular staining (eBioscience). All data were collected on a BD Fortessa LSRII (BD Biosciences) and analyzed with FlowJo software (TreeStar, Ashland, OR).

### Histological Analysis

The collected small intestine, and colon were fixed in 4% paraformaldehyde and embedded in paraffin. Sections (5 µm) were cut and stained with hematoxylin and eosin.

### Statistical Analyses

All the above data were analyzed by a two-tailed Student’s t-test with Graphpad Prism 5. P-values of <0.05 were considered statistically significant unless otherwise stated. All data were analyzed with Prism v8 software (GraphPad).

## Results

### The Abnormal Phenotype of ILC3s in ThPOK-Deficient Mice

**Figure 1:**
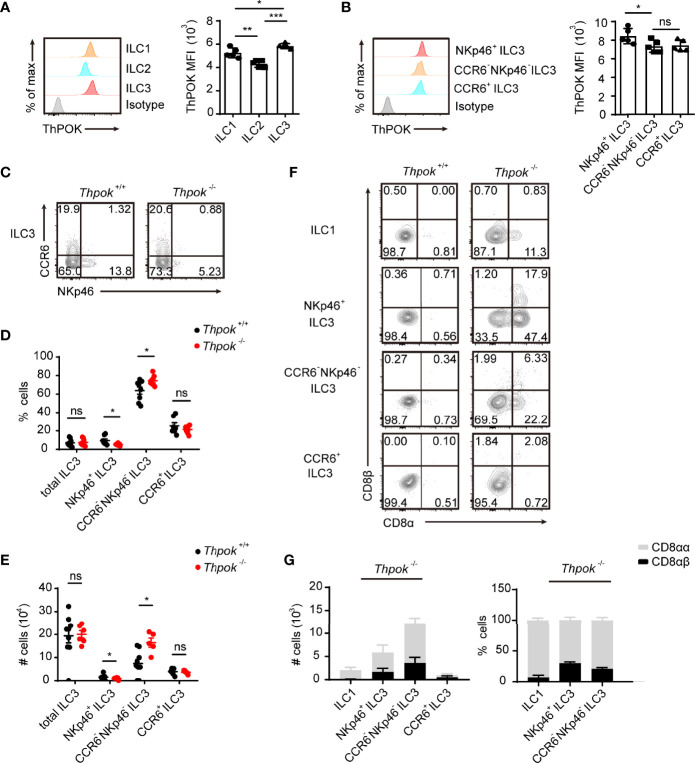
Defects in ILC3 differentiation in ThPOK-deficient mice. **(A)**Protein expression of ThPOK in ILC1s, ILC2s and ILC3s isolated from the siLP of C57BL/6N WT mice as measured by flow cytometry. ILCs were defined as Lineage- cells (lineage markers included TCRγδ, CD3ϵ, CD19, CD5, Gr-1 and Ter119). ILC1s were gated as Lin^-^ NKp1.1^+^ NKp46^+^ Eomes^-^ RORγt^-^. ILC2s were gated as Lin^-^ CD127^-^ KLRG1^+^ GATA3^+^. ILC3s were gated as Lin^-^ RORγt^+^. Representative histograms show ThPOK expression. The isotype controls are the shaded curves, ILC1s are orange curves, ILC2s are blue curves and ILC3s are red curves (left). Mean fluorescence intensity (MFI) of ThPOK in ILCs (right). ILCs were defined as Lineage^-^ cells (lineage markers included TCRγδ, CD3ϵ, CD19, CD5, Gr-1 and Ter119). (mean ± SEM; n = 5; *P < 0.05, **P < 0.01, ***P < 0.001, Student’s t test). **(B)** Representative histograms showing expression of ThPOK by different siLP ILC3 subsets (left). CCR6^+^ ILC3s were gated as Lin^-^ RORγt^+^ CCR6^+^ NKp46^-^. NKp46^+^ ILC3s were gated as Lin^-^ RORγt^+^ CCR6^-^ NKp46^+^. CCR6^-^ NKp46^-^ ILC3s were gated as Lin^-^ RORγt^+^ CCR6^-^ NKp46^-^. The red, orange and blue curves represent NKp46^+^, CCR6^-^ NKp46^-^ (DN) and CCR6^+^ ILC3s respectively. Mean fluorescence intensity (MFI) of ThPOK in three subpopulations (right). (mean ± SEM; n = 6-9; *P < 0.05, Student’s t test). **(C)** Flow cytometry assay of the percentages of ILC3s from the small intestine. ILC3 subsets were stained as Lin^-^RORγt^+^ then CCR6^+^ NKp46^-^, CCR6^-^ NKp46^-^, or CCR6^-^ NKp46^+^. Percentages **(D)** and total numbers **(E)** of different siLP ILC3 subsets. (mean ± SEM; n = 5-9; *P < 0.05, Student’s t test). **(F)** Flow cytometry analyzing CD8α and CD8β expression in ILC1s, NKp46^+^, CCR6^-^ NKp46^-^ (DN) and CCR6^+^ ILC3s. **(G)** Quantification of CD8αα and CD8αβ in indicated population from ThPOK-deficient mice. (mean ± SEM; n = 8; Student’s t test). Data are representative of at least three independent experiments. Statistical differences were tested using an unpaired Students’ t-test (two-tailed). ns, no significant difference.

We first investigated the expression level of ThPOK in three ILC subpopulations of the small intestinal lamina propria (siLP) using flow cytometry. Analysis showed that ThPOK was expressed in the three ILC groups and displayed the highest level in ILC3s compared to ILC1s or ILC2s (**Figure 1A**). Among all ILC3 subsets, the expression of ThPOK was slightly higher in NKp46^+^ ILC3s than in CCR6^+^ ILC3s and CCR6^-^ NKp46^-^ ILC3s (**Figure 1B**).

To further explore whether ThPOK participates in the differentiation and maintenance of intestinal ILCs, we analyzed the compositions of siLP ILCs in conventional ThPOK knockout mice. Phenotypic analysis revealed a selective loss of NKp46^+^ ILC3s in the absence of ThPOK, while the frequencies and total numbers of CCR6^+^ ILC3s stayed constant (**Figures 1C–E**). Subsequently, there was a marked increase in the ratio of CCR6^-^NKp46^-^ ILC3s, which are considered to be potential precursors of NKp46^+^ ILC3s. By contrast, the differentiation of ILC1s and ILC2s was not significantly changed by ThPOK deficiency (**Figures S1A–D**). These observations suggest that ThPOK is required for the maintenance of intestinal NKp46^+^ ILC3s and DN ILC3s.

To determine whether the reduced numbers of NKp46^+^ ILC3s were caused by increased apoptosis or decreased proliferation, we measured the levels of Annexin V and Ki67 in ThPOK-deficient ILC3s. The percentages of Annexin V^+^ and Ki67^+^ ILC3s of all subpopulations remained broadly unchanged in the absence of ThPOK (**Figures S1E, F**). We next tested ILC progenitors in the adult bone marrow to determine whether the variations in ILC3 subsets were due to alternations in progenitors of innate lymphoid cells in the bone marrow. Comparable numbers of cells were observed in the early development lineage for ILCs, such as common lymphoid progenitors (CLPs, Lin^-^CD127^+^c-Kit^int^Sca-1^int^Flt3^+^), α4β7^+^ lymphoid progenitors (αLPs, Lin^-^CD127^+^c-Kit^+^α4β7^+^), common helper-like innate lymphoid progenitors (CHILPs, Lin^-^CD127^+^α4β7^+^CD25^-^Flt3^-^), and common ILC precursors (ILCPs, Lin^-^c-Kit^+^CD127^+^α4β7^+^PLZF^+^) (**Figures S2A–D**). These observations suggest that ThPOK is not responsible for the early development of ILCs.

Considering that ThPOK has been reported to be required and sufficient for the repression of CD8 in NKT cells, ThPOK-deficient iNKT cells failed to express CD4, and a subset of them expressed low levels of CD8 (35). We explored whether ThPOK deficiency had a similar impact on ILCs. We found that ThPOK-deficient CCR6^+^ ILC3s expressed CD4 normally, and the percentages of CCR6^+^ CD4^+^ ILC3s and CCR6^+^ CD4^-^ ILC3s were comparable to those in control mice (**Figure S1G**). Thus, ThPOK disruption does not influence the continued CD4 expression in ILC3s. ILC3s typically do not express CD8, but approximately 25.7% of them re-expressed CD8α in deficient mice (**Figure S1H**). This change in phenotype was also seen in over 10% of the ILC1s but not in ILC2s, which displayed no expression of CD8α. Furthermore, ILC3s in ThPOK-deficient mice showed co-expression of CD8β (**Figure 1F**), especially in NKp46^+^ ILC3s, in which the expression was twice as high as that of CCR6^-^ NKp46^-^ ILC3s, yet there was little expression of CD8 in CCR6^+^ ILC3s (**Figures 1G**, **S1I, J**). Collectively, NKp46^+^ ILC3s showed the most significant change among the three subgroups, meaning that ThPOK deficiency is closely related to its function and development.

### Cell Intrinsic Regulation of NKp46^+^ ILC3 Development by ThPOK

**Figure 2:**
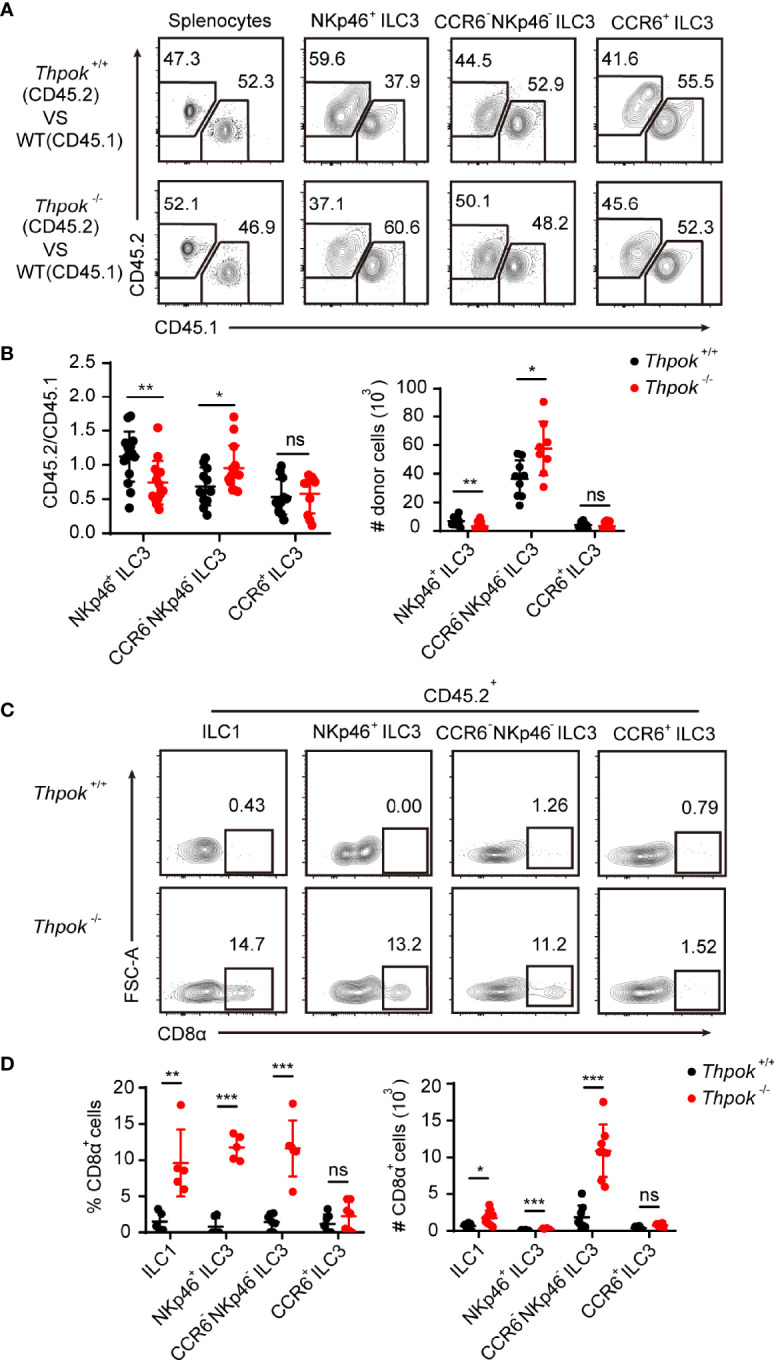
Abnormal differentiation of ILC3s in ThPOK-deficient mice is cell-intrinsic. **(A–D)** 2 × 10^6^ ThPOK-deficient or WT BM cells were co-transplanted with 2 × 10^6^ CD45.1 BM cells into lethally irradiated CD45.1 recipient mice. Examine donor chimerism of the indicated ILC3 subpopulations in small intestine 8 weeks later through flow cytometry. **(A)** Flow cytometric analysis of CD45.1 and CD45.2 expression in the indicated ILC3 subsets isolated from the siLP of reconstituted mice. Plots were gated on total splenocytes (left) and ILC3 subsets (right) respectively. **(B)** The percentages of donor derived cells in the indicated ILC3 subsets (CD45.2/CD45.1) were compared. The number of ThPOK donor derived cells (CD45.2^+^) in the indicated ILC3 subsets were shown on the right. (mean ± SEM; n = 8-13; *P < 0.05, **P < 0.01, Student’s t test). **(C)** Plots of CD8α expression in the indicated CD45.2^+^ cells were gated on total ILC1 (left) and ILC3 subsets (right) respectively. **(D)** Percentages and numbers of CD8α^+^ cells in the indicated cells were calculated. (mean ± SEM; n = 5-7; **P < 0.01, ***P < 0.001, Student’s t test). Data are representative of at least three independent experiments. ns, no significant difference.

Crosstalk between innate lymphoid cells and T cells has been described (36), and lineage commitment of T cells was also affected in ThPOK-deficient mice. Next, to verify that the effects of ThPOK deletion were cell-intrinsic, we generated competitive bone marrow chimaeras, where knockout and WT cells would compete in the same wild-type environment. We transplanted an equal ratio of CD45.1^+^ WT and CD45.2^+^ WT or ThPOK-deficient bone marrow cells into lethally irradiated CD45.1^+^ recipient mice. Donor chimaeras were sacrificed eight weeks after injection for further analysis of cells in spleen and intestinal lamina propria lymphocytes (LPL) based on Ly5.1 and Ly5.2 expression. ThPOK-sufficient and ThPOK-deficient bone marrow cells efficiently repopulated splenocytes at an equal ratio, proving the success of chimaera construction (**Figure 2A**). However, fewer NKp46^+^ ILC3s were derived from ThPOK-deficient donor cells along with a corresponding increase in the ratio of CCR6^-^ NKp46^-^ ILC3s compared to the WT competitor cells (**Figures 2A, B**). Importantly, no difference was detected in the percentage of CCR6^+^ ILC3s, corroborating the selective role of ThPOK in the homeostasis of CCR6^-^ ILC3s, including NKp46^+^ ILC3s and DN ILC3s.

We also detected CD8 expression in donor chimaeras. As expected, ILCs from WT donors remained CD8 negative, whereas ILC1s derived from ThPOK-deficient donor cells sustained CD8α expression (**Figure 2C**). Moreover, recipient mice reconstituted with ThPOK-deficient bone marrow cells had increased numbers of CD8α-expressing ILC3s. In line with our findings, NKp46^+^ ILC3s derived from knockout donor cells maintained the highest expression of CD8α among ILC3 subsets (**Figure 2D**). In summary, these findings demonstrate that ThPOK is required for proper repression of CD8 expression by ILC cells and for their development into NKp46^+^ ILC3s, which occurs in a cell-intrinsic manner.

### ThPOK Promotes the Function of Intestinal NKp46^+^ ILC3s Against *S. Typhimurium* Infection in A Cell intrinsic Manner

**Figure 3:**
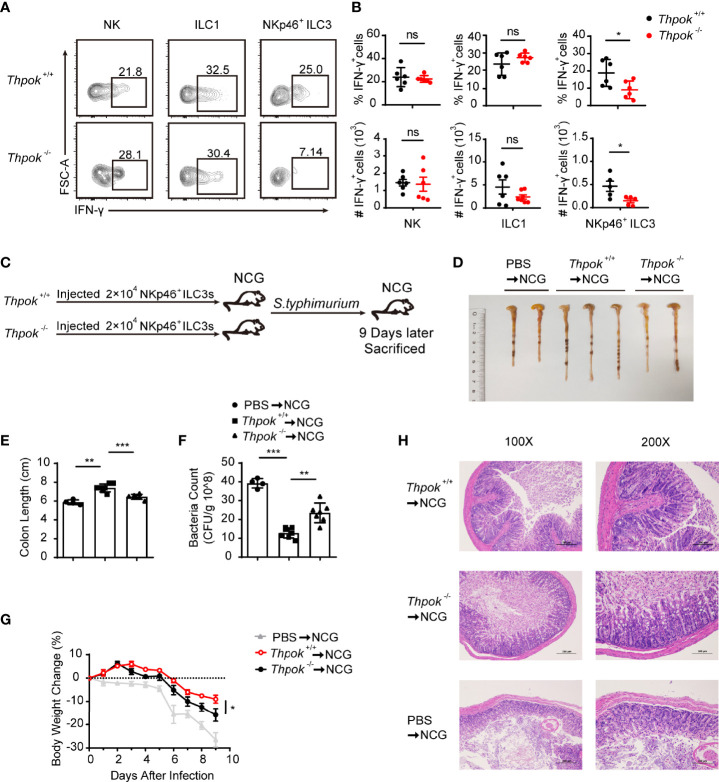
Defective function of NKp46^+^ ILC3s resulted in sensitivity to *S. typhimurium* infection after ThPOK deletion. **(A, B)** Cytokine production in siLP ILCs from ThPOK-deficient mice and their WT littermates. Cells were stimulated for 4 h *in vitro* with PMA and ionomycin for IFN-γ production. **(A)** Representative flow plots and **(B)** quantification of IFN-γ production from NK cells (left), ILC1s (middle), and NKp46^+^ ILC3s (right) after stimulation with PMA and ionomycin. (mean ± SEM; n = 5-6; *P < 0.05, Student’s t test). **(C)** NCG mice were adoptively transferred with 80000 intestinal NKp46^+^ ILC3s sorted from ThPOK-deficient mice and their WT littermates or PBS as control after treated with ABX for 1 week. NKp46^+^ ILC3s were injected to NCG mice through the tail vein. NCG mice were orally inoculated with *S. typhimurium* 24 hours post adoptive transfer. Body weight were monitored for 9 days, and all mice were sacrificed at day 9. **(D, E)** Colon length for NCG recipients after infection at day 9. (mean ± SEM; n = 4-7; **P < 0.01, ***P < 0.001, Student’s t test). **(F)** Bacterial counts in the feces at day 9 after infection. (mean ± SEM; n = 4-7; **P < 0.01, ***P < 0.001, Student’s t test). **(G)** Body weight changes at the indicated time points. (mean ± SEM; n = 6-11; *P < 0.05, Student’s t test). **(H)** Representative H&E staining of colon tissue sections. Data are representative of at least three independent experiments. ns, no significant difference.

In addition to the significant reduction in absolute numbers, we next explored whether the function of NKp46^+^ ILC3s was also affected. Therefore, we first tested the secretion of IFN-γ by NKp46^+^ ILC3s under stimulation conditions *in vitro*. We measured the intracellular IFN-γ expression in NK cells, ILC1s and NKp46^+^ ILC3s from siLP after stimulation with PMA and ionomycin for 4 hours. In contrast to the identical IFN-γ secretion of NK cells and ILC1s, we observed that IFN-γ-producing NKp46^+^ ILC3s were significantly reduced by 50% when compared to those in WT mice (**Figures 3A, B**). Group 1 ILCs and NKp46^+^ ILC3s help combat intracellular pathogens such as *Salmonella typhimurium* and viruses through IFN-γ secretion (37, 38). To specifically examine the effector function of NKp46^+^ ILC3s during infection and to rule out all influence from other immune cells, we isolated the equal number of highly purified NKp46^+^ ILC3s from control and ThPOK-deficient mice and adoptively transferred them into NCG (Rag2^-/-^ Il2rg^-/-^) mice, in which no ILCs or T/B cells exist. Then, we infected recipient mice with *S. Typhimurium* and monitored their body weight in the following days (**Figure 3C**). Nine days after infection, mice transferred with ThPOK-deficient NKp46^+^ ILC3s developed a more aggravated infection with concomitant significantly shorter colon lengths than the WT mice (**Figures 3D, E**). In addition to faster loss of body weight, higher colony counts were observed from the feces of NCG mice injected with ILC3s sorted from ThPOK-deficient mice (**Figures 3F, G**). Histological analysis of colons from infected ThPOK-deficient recipient mice showed more severe damage to the intestinal wall, including a significant increase in mucosal hyperplasia and submucosal inflammation compared to the control mice, implying compromised epithelial barrier function (**Figure 3H**). Collectively, these data indicate that ThPOK participates in the control of intestinal *S. typhimurium* infection by regulating IFN-γ production in NKp46^+^ ILC3s.

### ThPOK Participates in ILC3-Mediated Control of *C. rodentium* Infection by Regulating IL-17A Production

**Figure 4:**
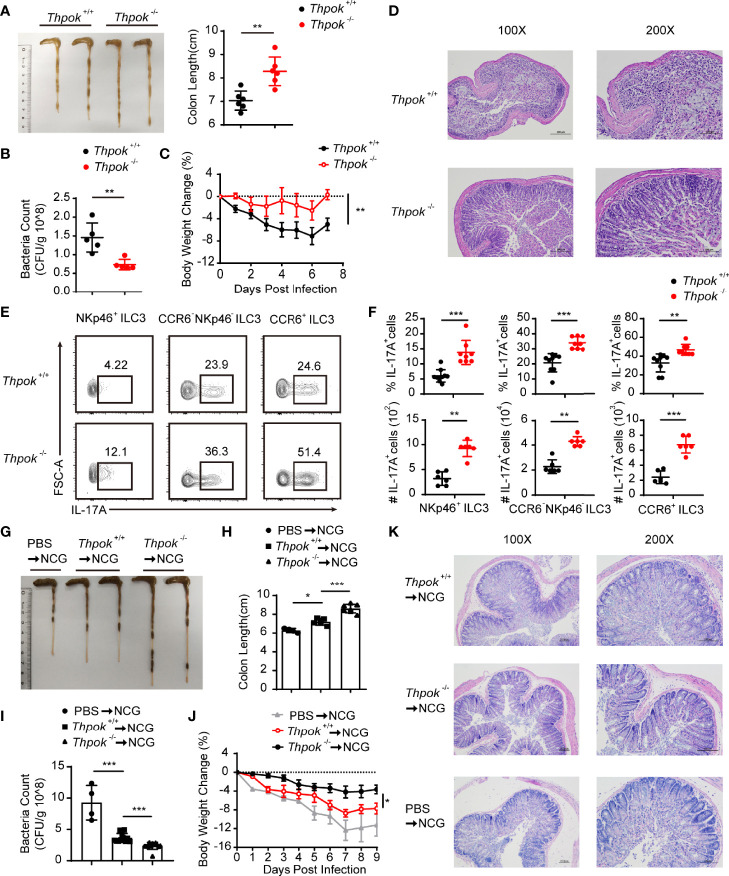
Decreased sensitivity to *C. rodentium* infection with IL-17A upregulation after ThPOK deletion. **(A–F)** ThPOK-deficient mice and their WT littermates were orally inoculated with 5 × 10^9^ CFUs of *C*. *rodentium* after treated with ABX for 1 week. Body weight were monitored for 7 days, and all mice were sacrificed at day 7. **(A)** Colons from ThPOK-deficient mice and their WT littermates were shown after infection at day 7 (left). Statistical analysis of the colon length (right). (mean ± SEM; n = 6; *P < 0.05, Student’s t test). **(B)** *C*. *rodentium* CFU in the feces on day 9 (mean ± SEM; n = 5; *P < 0.05, Student’s t test). **(C)** Body weight changes were monitored at the indicated time points. (mean ± SEM; n = 9; **P < 0.01, Student’s t test). **(D)** H&E staining of colon tissue sections was determined 7 days after infection. **(E, F)** Representative flow plots and quantification of IL-17A production from NKp46^+^ ILC3s (left), CCR6^-^ NKp46^-^ ILC3s (middle), and CCR6^+^ ILC3s (right) isolated from the siLP at day 7 after infection. (mean ± SEM; n = 6-9; **P < 0.01, ***P < 0.001, Student’s t test). **(G–K)** NCG mice were adoptively transferred with 80000 intestinal ILC3s sorted from ThPOK-deficient and WT mice or PBS as control after treated with ABX for 1 week. ILC3s were stimulated with IL-23 and IL-1β, at the concentration of 40 ng/ml, for 30min before injected to NCG mice through the tail vein. NCG mice were orally inoculated with *C*. *rodentium* 24 hours post adoptive transfer. Body weight were monitored for 9 days, and all mice were sacrificed at day 9. **(G, H)** Colon length for NCG recipients were measured and shown after *C*. *rodentium* infection at day 9. (mean ± SEM; n = 4-7; *P < 0.05, ***P < 0.001, Student’s t test). **(I)** Bacterial counts in the feces at day 9 after infection. (mean ± SEM; n = 4-11; ***P < 0.001, Student’s t test). **(J)** Body weight changes at the indicated time points. (mean ± SEM; n = 6-11; *P < 0.05, Student’s t test). **(K)** H&E staining of colon tissue sections was determined 9 days after infection. Data are representative of at least three independent experiments.

Studies have shown that ILC3s promote protective immunity against gastrointestinal infection and opportunistic fungal pathogens by producing IL-17A and IL-22. Therefore, we next tested the functionality of ThPOK-deficient ILC3s by assessing the production of effector cytokines upon *C. rodentium* infection. On Day 7 after infection, analysis of the gut in deficient mice showed longer colon lengths, together with lower bacterial titers in the intestine than control mice (**Figures 4A, B**). In line with the reduced bacterial dissemination in the colon, ThPOK-deficient mice showed a slower loss of body weight (**Figure 4C**). Following infection, the deficient mice had less persistent intestinal damage than their littermates, including features of less epithelial injury, decreased crypt hyperplasia, and weaker inflammatory cell infiltration (**Figure 4D**). These observations revealed a crucial role of ThPOK in ILC3s for host immunity against *C. rodentium* infection.

At an early stage of infection, IL-17A and IL-22 from group 3 innate lymphoid cells are required for limiting *C. rodentium* infection and enhancing host resistance (8, 19). We found that IL-17A-producing cells were strongly increased after infection, especially NKp46^+^ ILC3, the number of which was nearly double that in WT mice (**Figures 4E, F**). Similarly, CCR6^-^ NKp46^-^ ILC3s and CCR6^+^ ILC3s also exhibited increased IL-17A secretion in ThPOK-deficient mice. Nevertheless, these ThPOK-deficient mice showed similar percentages of IL-22-producing ILC3s during infection (**Figures S3A, B**).

IL-17A-mediated immunity has been identified as a pivotal host defense mechanism against fungal infections (39, 40). We then constructed a mouse *C. albicans* infection model to study the functional changes in ILC3s in ThPOK-deficient mice. Similarly, ThPOK-deficient mice infected with *C. albicans* displayed increased IL-17A production by ILC3 subpopulations (**Figures S4A, B**). Correspondingly, deficient mice exhibited efficient control of *C. albicans* infection, which was reflected by the longer colons (**Figures S4C, D**) and weakened bacterial dissemination compared with control mice (**Figure S4E**). In addition, deficient mice lost less body weight and exhibited less severe inflammation in HE-stained sections (**Figures S4F, G**).

Considering that other immune cells could also be affected in ThPOK-deficient mice, we purified intestinal ILC3s and adoptively transferred them into NCG mice. Nine days post-*C. rodentium* infection, colon length of mice injected with WT ILC3s was shorter than that of mice receiving ThPOK-deficient mice, but longer than that of mice without adoptive transfer (**Figures 4G, H**). Also, we collected feces at Day 9 after infection. Higher *C. rodentium* colony counts were observed from the feces of NCG mice injected with PBS than that of mice receiving WT ILC3s (**Figure 4I**). By contrast, NCG mice receiving ThPOK-deficient ILC3s had moderate levels of bacterial load in their feces. Compared with ThPOK-deficient cells, we found faster loss of body weight in the NCG mice receiving WT ILC3s and obvious manifestations of inflammatory damage such as decreased crypts, mucosal erosions, and infiltration of inflammatory cells (**Figures 4J, K**). These data demonstrate that the toxicity of *C. rodentium* is attenuated in mice with ThPOK-deficient cells and that the decreased sensitivity to *C. rodentium* infection occurs in a cell-intrinsic manner.

### Single-Cell RNA Sequencing Suggests ThPOK as a Driver of NKp46^+^ ILC3s Phenotypes

**Figure 5:**
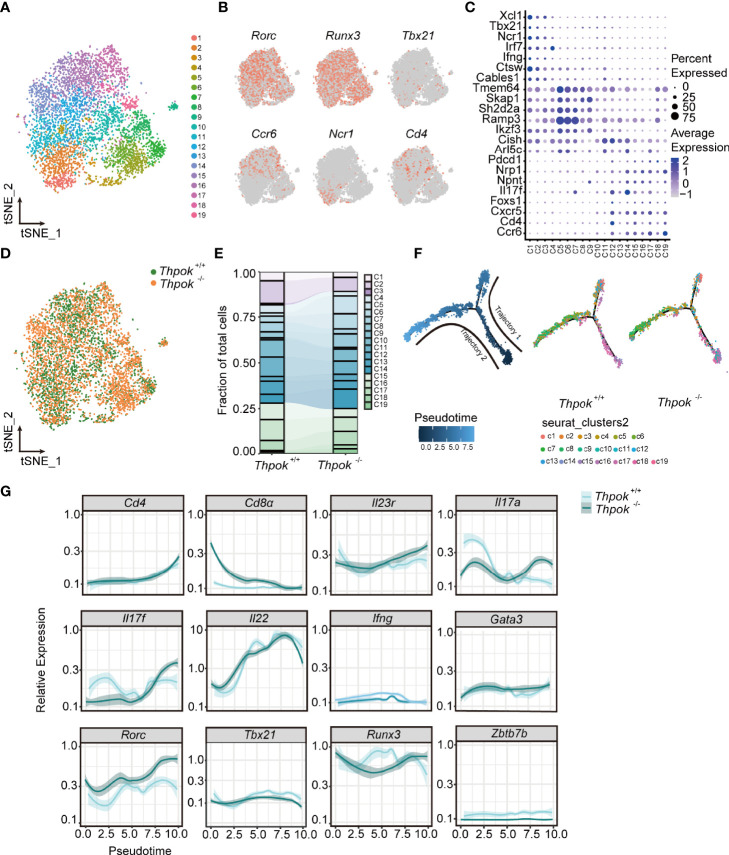
The transcriptomes profile of ILC3s from controls and ThPOK-deficient mice at a single-cellular level. **(A)** Biaxial t-SNE clustering plot of 6023 mixed cells from ThPOK-deficient and WT mice. Cells were grouped into 19 distinct clusters (C1-C19, colours indicated). **(B)** Distribution of key ILC regulators and cell markers shown by two-dimensional tSNE visualization. **(C)** Dot plot displaying expression of selected and previously described mice ILC subset-specific Transcription factors and cell surface markers used to annotate clusters. Average expression indicates normalized and scaled unique molecular identifiers (UMI). **(D)** tSNE plot showing the distribution of ThPOK-deficient and WT ILCs across all clusters, displayed as concatenated samples. **(E)** Quantitative analysis of WT and ThPOK-deficient cells across all clusters. Percentages of WT and ThPOK-deficient cells within each cluster among total ILC3 cells. **(F)** Calculated pseudotime trajectory based on differentially expressed genes in WT and deficient cells. The scale indicates the maturation state, from dark blue (least mature) to light blue (most mature). Cells are colored according to computed pseudotime coordinate, celltypes and clusters respectively, all clusters and individual cluster demonstrated in the trajectory. **(G)** Expression of involved key genes along the trajectory analysis.

To elucidate the mechanisms by which ThPOK regulates ILCs lineage maintenance, ILC3s were isolated from the small intestine and analyzed by single-cell RNA sequencing technology (BD Rhapsody). There were 6,647 valid cells in total (3,094 for WT mice and 3,553 for ThPOK-deficient mice), with a median of 1,500 gene transcripts per cell (**Figure S5A**). ILC3 cells were clustered for nineteen cellular compositions shown by t-distributed stochastic neighbor embedding (tSNE) (**Figure 5A**). The master transcription factor RAR-related orphan receptor gamma (RORγt; encoded by *Rorc*) was expressed across all clusters, assigning them to the ILC3 lineage (**Figure 5B**). Based on the reported ILC signature genes (41–44), we identified three layers with diverse transcriptional states. Cells in the bottle layer (clusters 1 to 3) constitutively expressed *Ncr1* and a small portion of cells in these clusters showed *Tbx21* expression (**Figure 5B**). Transcripts that were previously reported in NKp46^+^ ILC3s, including those encoding transcription factors such as *Ikzf3, Irf7* and genes encoding cell surface molecules such as *Ccr9, Fasl, Icos, Il12rb1*, and *Csf2*, were largely confined to the bottle layer (**Figures 5C**, **S5B**). The upper layer (clusters 15 to 19) was characterized by the expression of *Ccr6* and genes typically associated with CCR6^+^ ILC3s, such as *Cd4, Foxs1, Cxcr5, H2-oa, Pdcd1, Nrp1, S1pr1*, and *Il17f* but were negative for *Ncr1* and *Tbx21* (**Figures 5C**, **S5B**). ILC3s in the middle cell layer were negative for *Ccr6* and *Ncr1*, or other lineage-defining transcripts, but expressed *Il17rb, Chchd5, Hes1, Znrf3, Arl5c*, and *Zcchc18*.

All cells were also differentiated by mouse strain to show the alterations of cell clusters after ThPOK deletion (**Figure 5D**). The percentages of WT NKp46-specific layer cells were comparatively higher in clusters 1-3 (23% versus 13% in ThPOK-deficient mice), whereas ThPOK-deficient CCR6^-^NKp46^-^ cells were predominantly represented in cluster 5, 6, 8, 14 (nearly 65% versus 53% in WT mice). The average ratio of CCR6-specific layer cells was similar in both types (25% versus 22%) (**Figure 5E**). As shown in the figure, the pseudo-time trajectory revealed two distinct trajectories, along which wide-type and ThPOK-deficient ILC3s progressed differentially (**Figure 5F**). Trajectory 1 led through the cluster 6 toward clusters 1, 2, and 3 and was dominated by wide-type NKp46-specific layer cells. On the contrary, deficient CCR6-specific layer cells were analogous to WT CCR6-specific layer cells in trajectory 2, which led to ILC3 clusters 15 to 19 (**Figure 5F**). Cells on the trajectory exhibited an increase in *Rorc* expression along with downregulation of *Tbx21* expression (**Figure 5G**). Other key transcription factors involved in the interaction between *Rorc and Tbx21*were not influenced by ThPOK deletion, such as *Gata3*, *Runx3*, and *Ahr* (**Figures 5G**, **S5C**).

RORγt is important in the control of IL-23R expression and responsible for orchestrating the IL-23/IL-17 axis to promote inflammatory cytokine expression in innate lymphoid cells. In line with this, there were consistent increases in *Il23r*, *Il17a* and *Il17f* (**Figure 5G**). Both GO and KEGG analyses of RNA-seq data showed enrichment of DEGs associated with IL-17 secretion (e.g., IL-17 signaling pathway, cytokine-cytokine receptor interaction) (**Figure S5E**). Importantly, the expression of T-bet was substantially decreased by the loss of ThPOK and the same result was observed in the RNA-seq of ILC3s, including the decreased transcripts of *Notch2, Hes1*, and *Nr4a2* (**Figure S5C**). T-bet can directly regulate Notch2 in NKp46^+^ ILC3 differentiation. Insufficient T-bet results in lower Notch expression and blocks the transition of CCR6^-^NKp46^-^ ILC3 cells into NKp46^+^ ILC3s (45, 46), which partially impairs the cell pool of NKp46^+^ ILC3s in ThPOK-deficient mice. These data implied that ThPOK acted to repress the genes associated with IL-17-producing cells and to be a driver of NKp46^+^ ILC3s phenotypes.

### ThPOK Represses RORγt and IL23R Expression by Directly Binds to *Rorc* and *Il23r* in ILC3s

**Figure 6:**
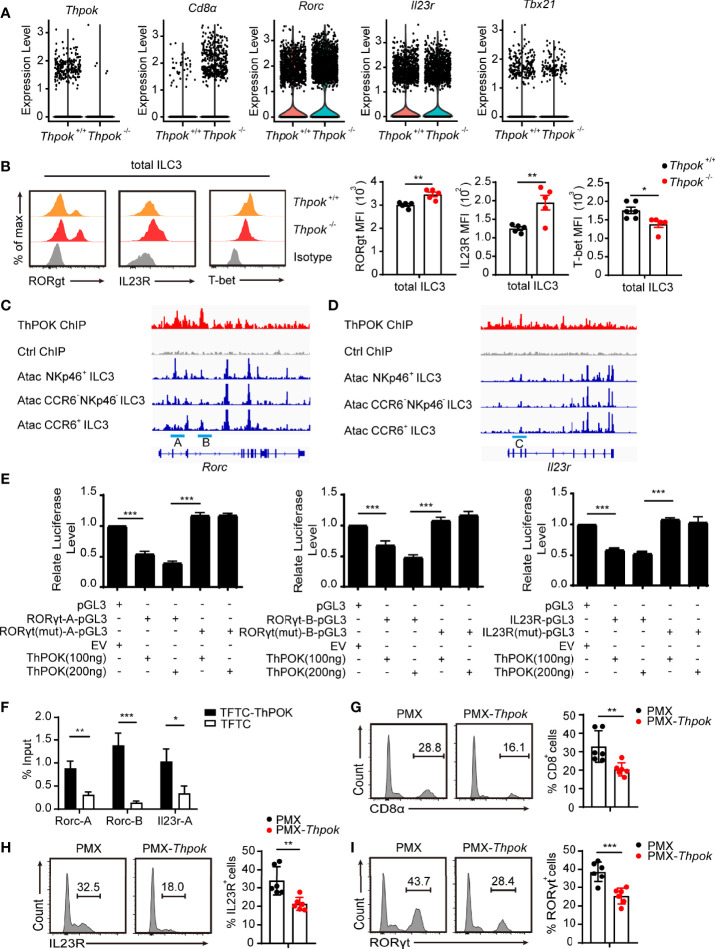
ThPOK negatively regulates the expression of RORγt and IL23R. **(A)**Violin plots showing differential expression of several ILC3 key genes. **(B)** Overlaid histograms (left) show expression of indicated protein in ThPOK-deficient mice (red), control mice (yellow) and Isotype (grey). Graphs (right) show MFI of protein expression. (mean ± SEM; n = 5; *P < 0.05, Student’s t test). **(C, D)** Graph of the *Rorc C*. and *Il23r* gene **(D)**, bottom tracks show Immgen ATAC-Seq peaks in NKp46^+^ ILC3s, DN ILC3s, and CCR6^+^ ILC3s (http://rstats.immgen.org/Chromatin/chromatin.html). Middle tracks show ChIPseq of activated CD4^+^ T cells from Zbtb7b^Bio/+^ Rosa26^BirA+^ (Thpok), or Zbtb7b^+/+^ Rosa26^BirA+^ (Ctrl) mice. **(E)** Luciferase activity in HEK293 cells transfected with various combinations (below plot) of vector alone (pGL3) or with 100, or 200 ng of vector containing the WT promoter of *Il23r*, *Rorc* or with mutations in the ThPOK-binding site of the promoter, together with empty vector (EV) or vector expressing ThPOK (ThPOK). The results are presented relative to cells transfected with pGL3 and empty vector (far left), set to one. (mean ± SEM; n = 6-9; ***P < 0.001, Student’s t test). **(F)** ThPOK binding at the *Il23r* and *Rorc* locus in EL4 stable cell lines expressing either a triple flag peptide (TFTC) or triple-flag-tagged ThPOK (TFTC-ThPOK) was monitored using a flag ChIP assay. The fold enrichment of ThPOK binding at each locus was normalized to TFTC-empty EL4 cells. (mean ± SEM; n = 6-9; *P < 0.05, **P < 0.01, ***P < 0.001, Student’s t test). **(G–I)** ILC3s were sorted from WT and ThPOK-deficient mice and infected with retrovirus containing the indicated vector. Flow cytometry for CD8, IL-23R and RORγt expression in ILC3s with the indicated vector overexpressed. (mean ± SEM; n = 6; **P < 0.01, ***P < 0.001, Student’s t test). Data are representative of at least three independent experiments.

Analysis revealed the abnormal expression of several key ILC3 genes, including upregulation of *Rorc*, *IL23r*, and downregulation of *Tbx21* (**Figure 6A**). We next tested whether the expression of these genes was different at the protein level in ILC3s from wide-type and ThPOK-deficient mice (**Figure 6B**). Validating our scRNA-seq data, flow cytometry profiles displayed comparably higher expression levels of RORγt and IL23R, along with lower expression levels of T-bet in ThPOK-deficient ILC3s compared to control mice (**Figure 6C**). Runx3 expression was comparable in the two strains (**Figures S5F–H**). The enhanced expression of RORγt and IL23R prompted us to figure out whether ThPOK was directly involved in their expression.

Several labs have identified the conserved ThPOK binding motif as GGGAGGG (47–49). We found several areas enriched for ThPOK binding within introns 2 and 3 of *Rorc* (site A and site B) by interrogating our recent mapping of Thpok DNA binding by ChIP-chip (**Figure 6C**) (50). ChIP-seq also confirmed the presence of a ThPOK binding peak within the 5’ half of intron 2 of *IL23r*, named site C (**Figure 6D**). These three regions were also found to contain ATAC-seq peaks identifying areas of accessible chromatin (51). To examine whether ThPOK can directly affect the expression of IL23R and RORγt with the suggested ThPOK-binding site, we inserted three sites in luciferase reporter vectors and evaluated their luciferase reporter activity by transfection experiments in EL4 cells. We observed that after the addition of ThPOK expression vector, the luciferase activity was repressed and there was a significant decrease in the luciferase activity of EL4 cells transfected with vector containing sites A, B, and C compared with cells transfected with the empty vector (**Figure 6E**). Furthermore, deletion of the ThPOK-binding motif in *Il23r* and *Rorc* abrogated the reduction of luciferase activity (**Figure 6E**). These results demonstrated that ThPOK negatively regulates the expression of IL23R and RORγt in a manner dependent on ThPOK-binding motifs.

To further elucidate the mechanism by which ThPOK regulates the expression of IL23R and RORγt, we performed ChIP assays in the EL4 cell line, which overexpressed the ThPOK-flag plasmid. The results of ChIP-PCR verified ThPOK binding to the regions (**Figure 6F**). We next rescued the increased expression of CD8, IL23R and RORγt *via* overexpression of ThPOK in group 3 ILCs from ThPOK-deficient mice. After overexpression, ThPOK reconstituted cells had decreased expression of CD8, IL23R and RORγt (**Figures 6G–I**). These findings further identified the regions of *Il23r* and *Rorc* that both bound and functionally responded to ThPOK.

## Discussion

Although several studies have uncovered the role of ThPOK in predetermining the lineage commitment of T cell population, as well as natural killer T cells and γδ T cells, its function in ILC development remines largely unknown. In this study, we show that ThPOK is expressed in three groups of ILCs. ThPOK deficiency impairs the maintenance and IFNγ secretion of NKp46^+^ ILC3s, resulting in the sensitivity to *S. typhimurium*. Additionally, ILC1s and ILC3s re-expressed CD8α, whereas no alterations in CD4 were observed in deficient mice. Moreover, ThPOK participates in ILC3-mediated control of *C. rodentium* infection by negatively regulating IL-17A secretion. The deficient ILC3s exhibited increased levels of RORγt and IL23R, which further promoted IL-17A secretion.

Recent comparative transcriptome analyses have revealed defined transcription factors involved in ILC lineage commitment, such as T-bet, RORγt, and GATA-3. Graded expression of T-bet determines the fate of CCR6^-^ NKp46^+^ ILC3s (14). In CCR6^-^ NKp46^+^ ILC3s, T-bet expression acts as a molecular switch inducing the transition from ILC3 to ILC1. High level of T-bet expression can also serve as a repressor of RORγt, eventually resulting in a full conversion from a type 3 to a type 1 phenotype. Recent studies demonstrated that ILC3-specific RORγt antagonized the regulator T-bet in NKp46^+^ ILC3s (26), indicating an antagonism between T-bet and RORγt (52, 53). Besides, GATA-3 acts upstream of T-bet and regulates the balance between RORγt and T-bet, which is critical for specifying the NKp46^+^ lineage fate. Similarly, we found that mice lacking ThPOK exhibit marked upregulation of RORγt, which prevents the expression of T-bet and, consequently, T-bet target genes. Corresponding to reduced T-bet, the development of NKp46^+^ ILC3s and IFN-γ production was abolished. As a critical transcription factor for CD4 fate determination in T cells upstream of ThPOK, GATA-3 can directly regulate the expression of ThPOK (54, 55). We may speculate that ThPOK might function as a downstream target gene of GATA-3, which induces the interplay between RORγt and T-bet. Different from NKp46^+^ ILC3 cells, CCR6^+^ (CD4^+/−^) ILC3 cells represent a separate population, which are thought to have a distinct progenitor. Although RORγt^+^ LTi progenitors express low amounts of GATA-3, GATA-3 is only required for the development of PLZF-expressing non-LTi progenitors (56). As supposed, as a downstream target gene of GATA-3, deletion of ThPOK showed no effect on the development of LTi-like progenitors, offering a plausible explanation for unchanged development of CCR6^+^ ILC3s (CD4^+^ ILC3s and CD4^-^ ILC3s) in ThPOK deficient mice.

In addition to its effect on ILC3 homeostasis and NKp46^+^ ILC3 subset development, ThPOK also regulates the expression of the critical effector cytokine IL-17A. The production of IL-17 is usually under the control of the transcription factor RORγt (29), which controls IL-23R expression and further influences the IL-23/IL-17 axis. Of note, RORγt expression is downstream of Runx3 in ILC3s. Runx3 can directly regulate RORγt expression in a Runx3-binding motif-specific manner (29). ThPOK can directly bind to *Rorc* for proper repression of RORγt expression, whereas Runx3 promotes the expression of RORγt and its downstream target genes (29). We may reasonably speculate that ThPOK and Runx3 antagonize each other in RORγt regulation and directly disturb IL-17A production. Another key marker, IL23R, which is responsible for IL-17 production, was also increased in deficient mice. The enhanced IL23R expression further contributes to the secretion of IL-17A by ILC3s. The transcription factor Runx3 is also critical for the development of the CD8^+^ lineage by activating CD8 and inhibiting ThPOK (4, 14), whereas CD8 expression was stably repressed by ThPOK *via* direct suppression of multiple enhancers (47, 57). We supposed that after ThPOK deletion, the inhibitory effect of CD8 in ILC1s and ILC3s was lifted, allowing the re-expression of CD8 molecules with Runx3. Overall, our study demonstrates the pivotal role of ThPOK in modulating the development, and functions of ILC3 subsets and adds ThPOK as a novel key factor to the list of the transcriptional network effectors that guides the differentiation of ILCs as a regulator of NKp46^+^ ILC3s.

## Data Availability Statement

This original data presented in the study can be found here: https://www.ncbi.nlm.nih.gov/geo/query/acc.cgi?acc=GSE203422 and GSE204698 and available from the corresponding authors upon reasonable request.

## Ethics Statement

The animal study was reviewed and approved by Ethics Committee of Zhejiang University.

## Author Contributions

Conceptualization, LW, LL. Data curation, XG, XS, KL, CL, YF, QX, ZH. Methodology, XG, XS, KL, CL, YF, QX, ZH. Visualization, XG, XS, CL, YF, QX, ZH, XL. Software, KL. Resources, SH. Funding acquisition, XM. Writing - original draft, XG. Supervision, LW. Writing - review and editing, LW. All authors contributed to the article and approved the submitted version.

## Funding

This work was supported by grants from the National Natural Science Foundation of China (32030035, 31870874, 32000623, 32100693), Zhejiang Provincial Natural Science Foundation (LZ21C080001).

## Conflict of Interest

The authors declare that the research was conducted in the absence of any commercial or financial relationships that could be construed as a potential conflict of interest.

## Publisher’s Note

All claims expressed in this article are solely those of the authors and do not necessarily represent those of their affiliated organizations, or those of the publisher, the editors and the reviewers. Any product that may be evaluated in this article, or claim that may be made by its manufacturer, is not guaranteed or endorsed by the publisher.

## Supplementary Material

Supplementary Figure 1 ILCs’ Phenotype in ThPOK-deficient mice. **(A)** Flow cytometry analysis of NK and ILC1s from siLP. Group 1 ILC cells from siLP were gated as Lin^-^/NK1.1^+^/NKp46^+^/RORγt^-^ cells and then analyzed as NK cells (defined as Lin^-^/NK1.1^+^/NKp46^+^/Eomes^+^/RORγt^-^) and ILC1 cells (defined as Lin^-^/NK1.1^+^/NKp46^+^/Eomes^-^/RORγt^-^). ILC2s from siLP were gated as Lin^-^/KLRG1^+^/GATA3^+^. **(B)** Flow cytometry analysis of ILC2s from siLP. **(C, D)** Percentages and cell numbers of NK, ILC1s and ILC2s are represented in the right. (mean ± SEM; n = 6-8; Student’s t test). **(E)** Proliferation of the indicated ILC3 subsets labeled with ki67 and isolated from the siLP of ThPOK-deficient mice and their WT littermates (upper). Graphs below show percentages of Ki67^+^ cells. (mean ± SEM; n = 5; Student’s t test). **(F)** Apoptosis of indicated ILC3 subsets labeled with Annexin V (upper). Graphs below show quantification of Annexin V^+^ cells. (mean ± SEM; n = 5; Student’s t test). **(G)** Flow cytometry analysis of CD4^+^ cells in the siLP, stained with RORγt, CD4 and CCR6 (left). Frequencies of CCR6^+^ CD4^-^ ILC3s and CCR6^+^ CD4^+^ ILC3s (right). (mean ± SEM; n = 7-9; Student’s t test). **(H)** Frequencies of CD8α positive ILC1s, ILC2s and ILC3s from siLP of ThPOK-deficient mice and their WT littermates as measured by flow cytometry. **(I)** Expression of CD8α by different siLP ILC3 subsets from ThPOK-deficient mice and their WT littermates. Representative histograms (upper) show CD8α expression. Graphs below show quantification of CD8α gMFI (mean ± SEM; n = 7-8; *******p<0.001, Student’s t test). **(J)** Expression of CD8β by different siLP ILC3 subsets from ThPOK-deficient mice and their WT littermates. Representative histograms (upper) show CD8α expression. Graphs below show quantification of CD8α gMFI (mean ± SEM; n = 7-8; *******p<0.001, Student’s t test). Data are representative of at least three independent experiments.

Supplementary Figure 2 Loss of ThPOK does not disturb ILC precursors formation in Bone Marrow. **(A–D)** Frequencies and absolute numbers of common lymphoid progenitor cells (CLPs, Lin^-^/CD127^+^/c-Kit^int^/Sca1^int^/Flt3^+^), common progenitor to all helper-like innate lymphoid cell lineages (ChILPs, Lin^-^/CD127^+^/α4β7^+^/CD25^-^/Flt3^-^), α4β7 integrin-expressing CLP (α-LPs, Lin^-^/CD127^+^/c-Kit^+^/α4β7^+^), and common precursor to ILC (ILCPs, Lin^-^/CD127^+^/α4β7^+^/PLZF^+^) in bone marrow of ThPOK-deficient mice and their WT littermates. (mean ± SEM; n = 5; Student’s t-test). Data are representative of at least three independent experiments.

Supplementary Figure 3 The secretion of IL-22 remains unchanged in *C. rodentium* Infection. **(A, B)** Representative flow plots and quantification of IL-22 production from total ILC3s, NKp46^+^ ILC3s, CCR6^-^ NKp46^-^ ILC3s, and CCR6^+^ ILC3s isolated from the siLP at day 7 after *C. rodentium* infection. (mean ± SEM; n = 6-9; Student’s t test). Data are representative of at least three independent experiments.

Supplementary Figure 4 ThPOK promotes intestinal ILC3s function against *C. albicans* infection. **(A–G)** ThPOK-deficient mice and their WT littermates were orally inoculated with 1 × 10^8^ CFUs of *C. albicans* (SC5314). Body weight changes were monitored for 7 days, and all mice were sacrificed at day 7. **(A, B)** Representative flow plots and quantification of IL-17A production from toal ILC3s and three subpopulations at day 7 post infection. (mean ± SEM; n = 5-6; *****P < 0.05, ******P < 0.01, *******P < 0.001, Student’s t test). **(C, D)** Colons from ThPOK-deficient mice and their WT littermates were shown and measured at day 7 post infection. (mean ± SEM; n = 8-9; ******P < 0.01, Student’s t test). **(E)** Fecal bacterial counts at post infection day 7. (mean ± SEM; n = 6; ******P < 0.01, Student’s t test). **(F)** Body weight changes were shown at the indicated time points. (mean ± SEM; n = 6-9; *****P < 0.05, Student’s t test). **(G)** H&E histological analysis of representative colons from infected mice at post-infection day 7. Data are representative of at least three independent experiments.

Supplementary Figure 5 Analysis of differentially expressed genes associated with ILC3s in controls and ThPOK-deficient mice. **(A)** The numbers of the median numbers of genes that passed quality filtering. **(B)** Feature plots of expression distribution for transcription factors, cell surface markers and cytokines across pseudo-time. **(B)** Dot plot displaying differentially expressed genes across all clusters. Top 10 up-regulated DEGs (ranked by log fold change) of each cluster were plotted using heatmap. **(C)** Expression of involved key genes along the trajectory analysis. **(D)** The Volcano plot of upregulated and downregulated genes in WT and ThPOK-deficient ILC3s of the RNA-seq data set. **(E)** KEGG pathway enrichment analysis performed by using DAVID. The top 15 highly enriched KEGG pathways are presented. KEGG, Kyoto Encyclopedia of Genes and Genomes database; DAVID, Database for Annotation, Visualization and Integrated Discovery. **(F)**Violin plots showing differential expression of several ILC3 key genes. **(G)** Overlaid histograms (upper) show expression of indicated protein in ThPOK-deficient mice(red), control mice (yellow) and Isotype (grey). Graphs (lower) show MFI of protein expression. **(H)** Mean fluorescence intensity (MFI) of the indicated proteins in ILC3s. (mean ± SEM; n = 5-8; Student’s t test). Data are representative of at least three independent experiments.

## Abbreviations

DN ILC3s, CCR6^-^ NKp46^-^ ILC3s; ILC, innate lymphoid cell; siLP, small intestinal lamina propria; ThPOK, T-helper-inducing POZ-Kruppel Factor; WT, wild-type.
