# Supplementary figures and images for: The Transcription Factor ThPOK Regulates ILC3 Lineage Homeostasis and Function During Intestinal Infection
Source: Front Immunol. 2022 Jul 1;13:939033. doi: 10.3389/fimmu.2022.939033 (PMC9285022; doi:10.3389/fimmu.2022.939033)

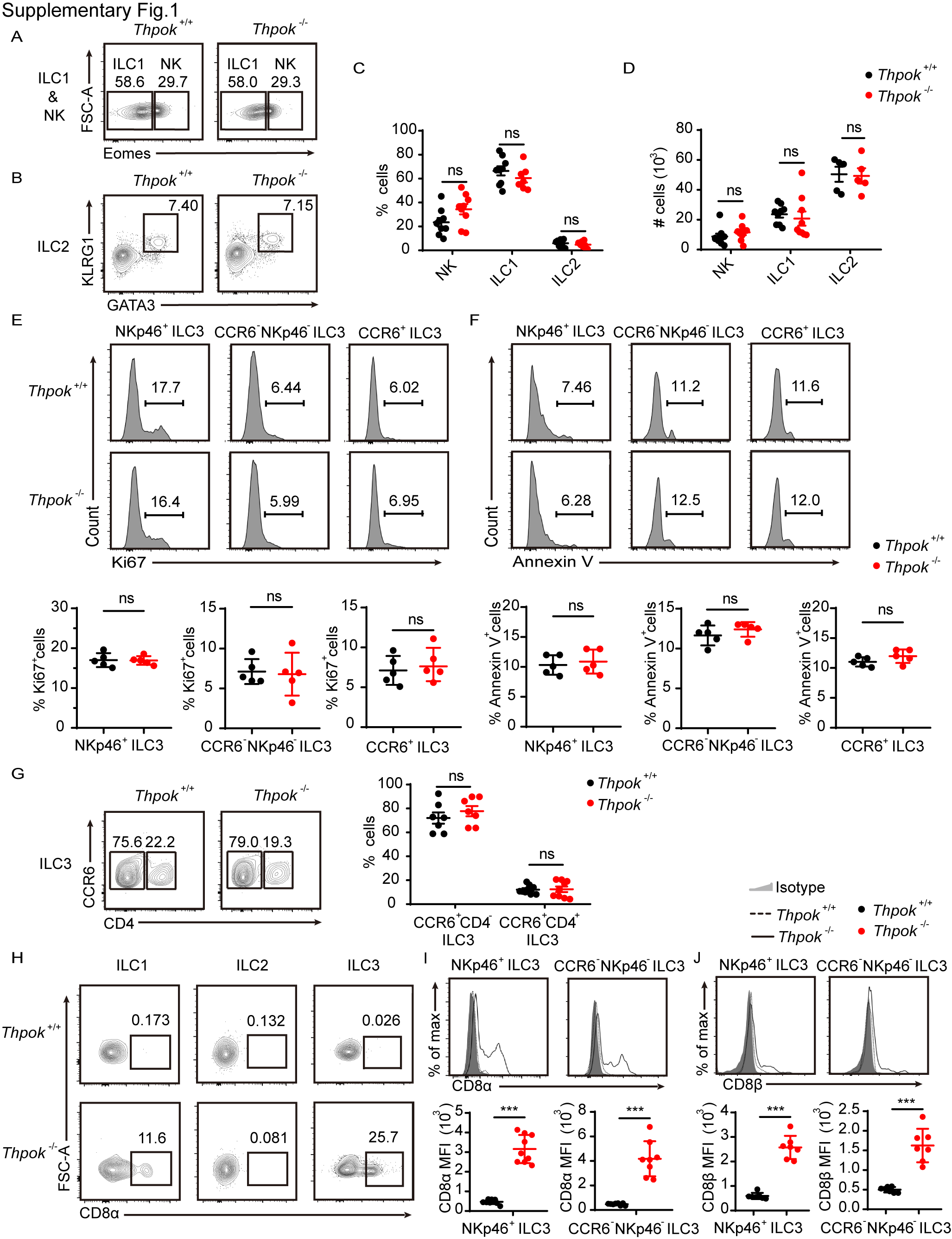

Supplement: Supplementary Figure 1 — ILCs’ Phenotype in ThPOK-deficient mice. (A) Flow cytometry analysis of NK and ILC1s from siLP. Group 1 ILC cells from siLP were gated as Lin-/NK1.1+/NKp46+/RORγt- cells and then analyzed as NK cells (defined as Lin-/NK1.1+/NKp46+/Eomes+/RORγt-) and ILC1 cells (defined as Lin-/NK1.1+/NKp46+/Eomes-/RORγt-). ILC2s from siLP were gated as Lin-/KLRG1+/GATA3+. (B) Flow cytometry analysis of ILC2s from siLP. (C, D) Percentages and cell numbers of NK, ILC1s and ILC2s are represented in the right. (mean ± SEM; n = 6-8; Student’s t test). (E) Proliferation of the indicated ILC3 subsets labeled with ki67 and isolated from the siLP of ThPOK-deficient mice and their WT littermates (upper). Graphs below show percentages of Ki67+ cells. (mean ± SEM; n = 5; Student’s t test). (F) Apoptosis of indicated ILC3 subsets labeled with Annexin V (upper). Graphs below show quantification of Annexin V+ cells. (mean ± SEM; n = 5; Student’s t test). (G) Flow cytometry analysis of CD4+ cells in the siLP, stained with RORγt, CD4 and CCR6 (left). Frequencies of CCR6+ CD4- ILC3s and CCR6+ CD4+ ILC3s (right). (mean ± SEM; n = 7-9; Student’s t test). (H) Frequencies of CD8α positive ILC1s, ILC2s and ILC3s from siLP of ThPOK-deficient mice and their WT littermates as measured by flow cytometry. (I) Expression of CD8α by different siLP ILC3 subsets from ThPOK-deficient mice and their WT littermates. Representative histograms (upper) show CD8α expression. Graphs below show quantification of CD8α gMFI (mean ± SEM; n = 7-8; ***p<0.001, Student’s t test). (J) Expression of CD8β by different siLP ILC3 subsets from ThPOK-deficient mice and their WT littermates. Representative histograms (upper) show CD8α expression. Graphs below show quantification of CD8α gMFI (mean ± SEM; n = 7-8; ***p<0.001, Student’s t test). Data are representative of at least three independent experiments. [file Image_1.tif]

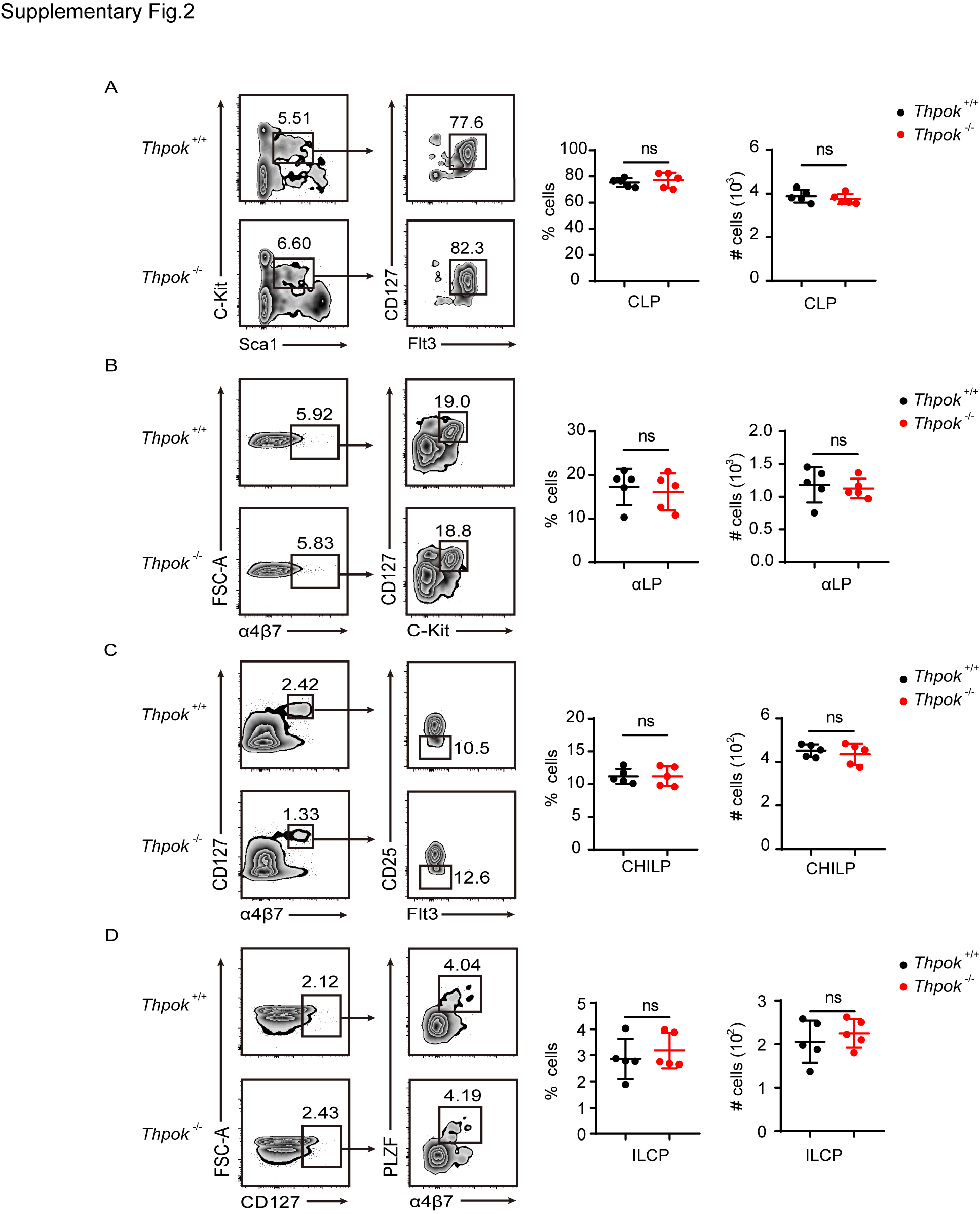

Supplement: Supplementary Figure 2 — Loss of ThPOK does not disturb ILC precursors formation in Bone Marrow. (A–D) Frequencies and absolute numbers of common lymphoid progenitor cells (CLPs, Lin-/CD127+/c-Kitint/Sca1int/Flt3+), common progenitor to all helper-like innate lymphoid cell lineages (ChILPs, Lin-/CD127+/α4β7+/CD25-/Flt3-), α4β7 integrin-expressing CLP (α-LPs, Lin-/CD127+/c-Kit+/α4β7+), and common precursor to ILC (ILCPs, Lin-/CD127+/α4β7+/PLZF+) in bone marrow of ThPOK-deficient mice and their WT littermates. (mean ± SEM; n = 5; Student’s t-test). Data are representative of at least three independent experiments. [file Image_2.tif]

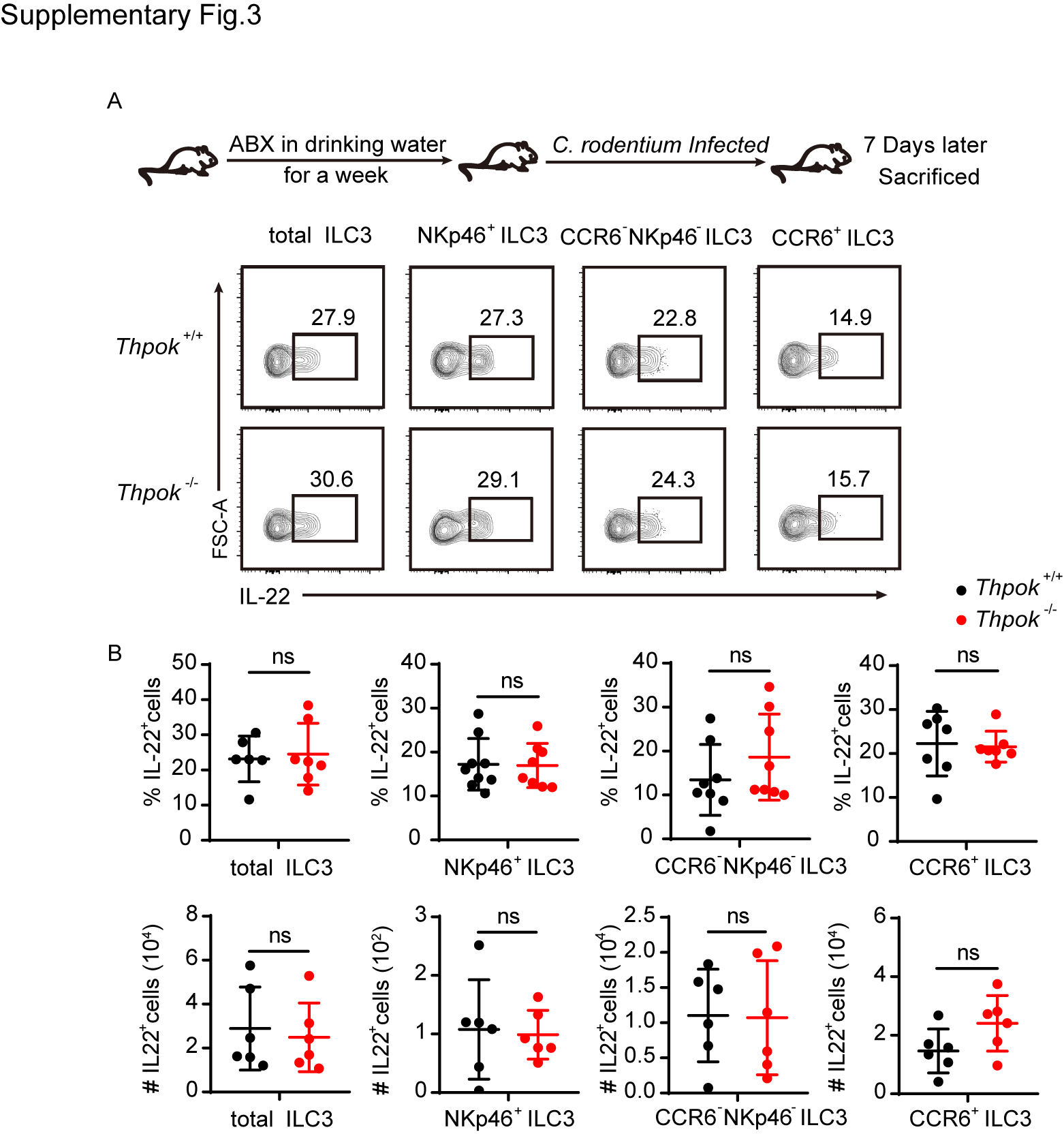

Supplement: Supplementary Figure 3 — The secretion of IL-22 remains unchanged in C. rodentium Infection. (A, B) Representative flow plots and quantification of IL-22 production from total ILC3s, NKp46+ ILC3s, CCR6- NKp46- ILC3s, and CCR6+ ILC3s isolated from the siLP at day 7 after C. rodentium infection. (mean ± SEM; n = 6-9; Student’s t test). Data are representative of at least three independent experiments. [file Image_3.tif]

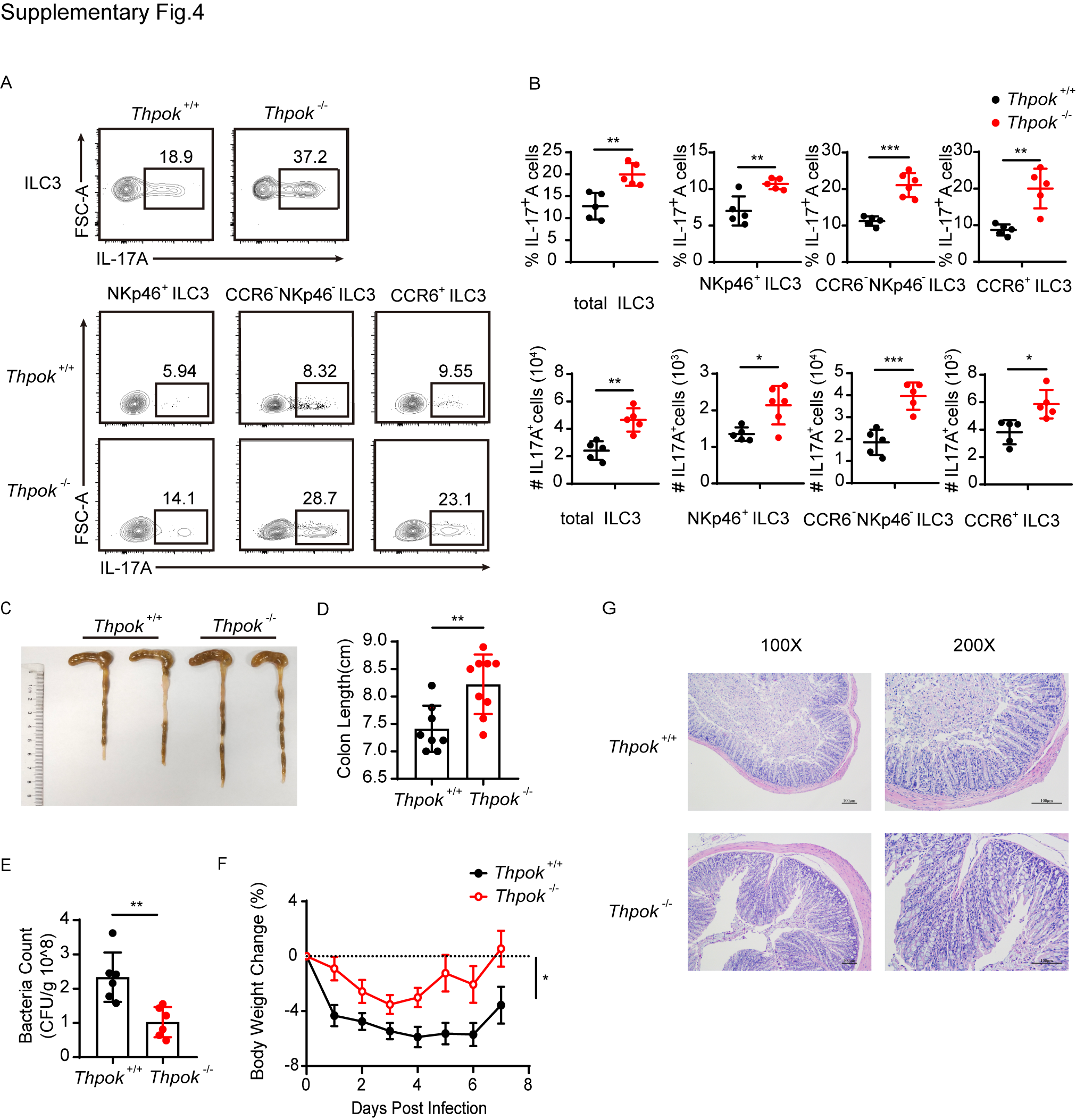

Supplement: Supplementary Figure 4 — ThPOK promotes intestinal ILC3s function against C. albicans infection. (A–G) ThPOK-deficient mice and their WT littermates were orally inoculated with 1 × 10^8^ CFUs of C. albicans (SC5314). Body weight changes were monitored for 7 days, and all mice were sacrificed at day 7. (A, B) Representative flow plots and quantification of IL-17A production from toal ILC3s and three subpopulations at day 7 post infection. (mean ± SEM; n = 5-6; *P < 0.05, **P < 0.01, ***P < 0.001, Student’s t test). (C, D) Colons from ThPOK-deficient mice and their WT littermates were shown and measured at day 7 post infection. (mean ± SEM; n = 8-9; **P < 0.01, Student’s t test). (E) Fecal bacterial counts at post infection day 7. (mean ± SEM; n = 6; **P < 0.01, Student’s t test). (F) Body weight changes were shown at the indicated time points. (mean ± SEM; n = 6-9; *P < 0.05, Student’s t test). (G) H&E histological analysis of representative colons from infected mice at post-infection day 7. Data are representative of at least three independent experiments. [file Image_4.tif]

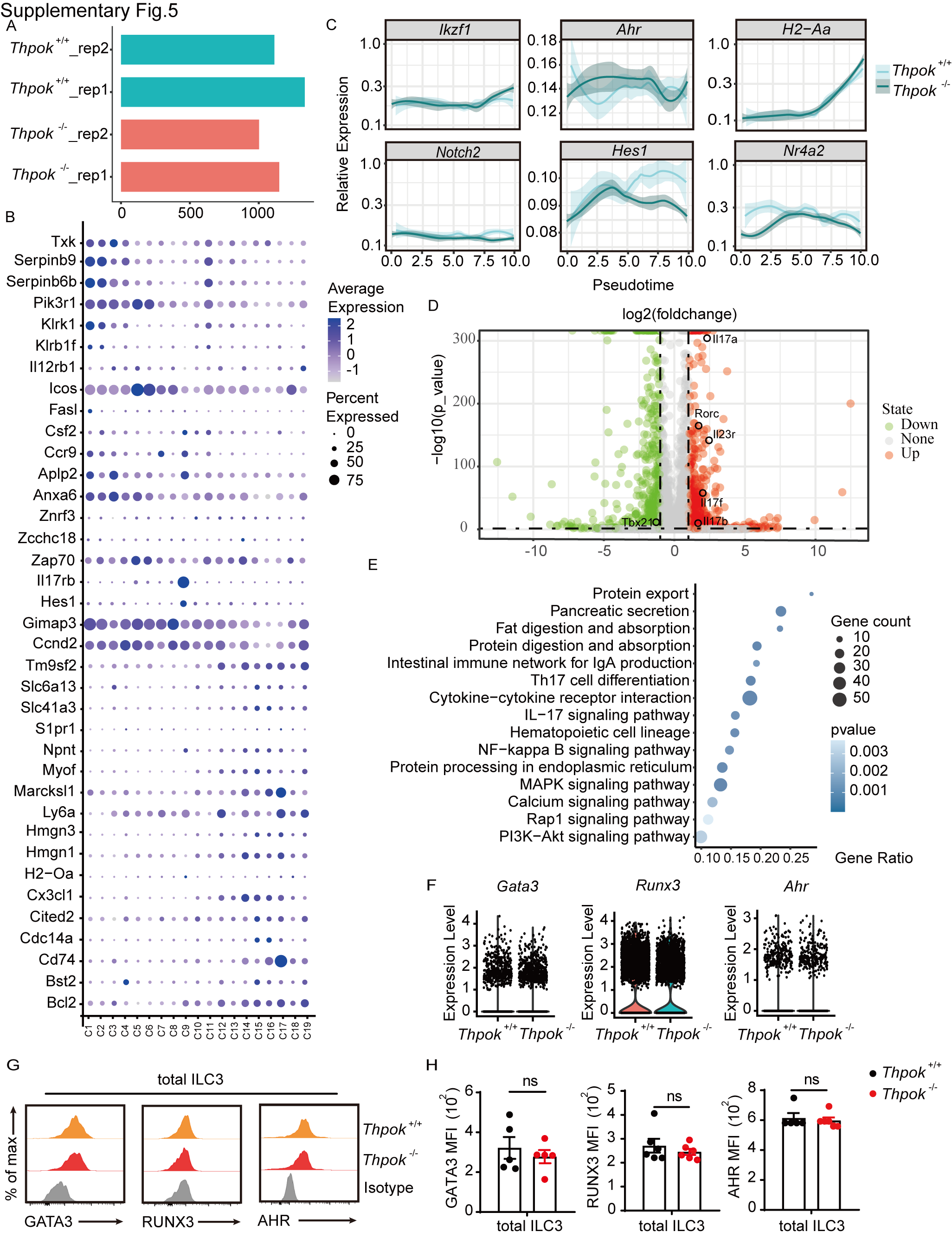

Supplement: Supplementary Figure 5 — Analysis of differentially expressed genes associated with ILC3s in controls and ThPOK-deficient mice. (A) The numbers of the median numbers of genes that passed quality filtering. (B) Feature plots of expression distribution for transcription factors, cell surface markers and cytokines across pseudo-time. (B) Dot plot displaying differentially expressed genes across all clusters. Top 10 up-regulated DEGs (ranked by log fold change) of each cluster were plotted using heatmap. (C) Expression of involved key genes along the trajectory analysis. (D) The Volcano plot of upregulated and downregulated genes in WT and ThPOK-deficient ILC3s of the RNA-seq data set. (E) KEGG pathway enrichment analysis performed by using DAVID. The top 15 highly enriched KEGG pathways are presented. KEGG, Kyoto Encyclopedia of Genes and Genomes database; DAVID, Database for Annotation, Visualization and Integrated Discovery. (F)Violin plots showing differential expression of several ILC3 key genes. (G) Overlaid histograms (upper) show expression of indicated protein in ThPOK-deficient mice(red), control mice (yellow) and Isotype (grey). Graphs (lower) show MFI of protein expression. (H) Mean fluorescence intensity (MFI) of the indicated proteins in ILC3s. (mean ± SEM; n = 5-8; Student’s t test). Data are representative of at least three independent experiments. [file Image_5.tif]
